# Underprescription of Clozapine: A Narrative Review Regarding ‘Clozaphobia’

**DOI:** 10.1002/hup.70050

**Published:** 2026-05-14

**Authors:** Rafael Brito Castro, Joaquim J. Ferreira, João Gama‐Marques

**Affiliations:** ^1^ Clínica Universitária de Psiquiatria e Psicologia Médica (CUPPM) Faculdade de Medicina Universidade de Lisboa (FMUL) Centro Académico de Medicina de Lisboa (CAML) Lisboa Portugal; ^2^ Laboratório de Farmacologia Clínica e Terapêutica (LFCT) Faculdade de Medicina, Universidade de Lisboa (FMUL), Centro Académico de Medicina de Lisboa (CAML) Lisboa Portugal; ^3^ Campus Neurológico Sénior (CNS) Torres Vedras Portugal; ^4^ Consulta de Esquizofrenia Resistente (CER) Hospital de Júlio de Matos (HJM), Unidade Local de Saúde de São José (ULSSJ), Centro Clínico Académico de Lisboa (CCAL) Lisboa Portugal

**Keywords:** clozaphobia, clozapine, resistant, schizophrenia, treatment

## Abstract

This review aims to examine the appropriateness of the term ‘clozaphobia’ to characterise the obstacles contributing to the underprescription of clozapine (CLZ). English‐language, peer‐reviewed studies addressing clinicians', patients' and/or carers' attitudes toward CLZ were considered eligible for full‐text review. In May 2025, a search was conducted using the keywords *clozapine, clozaphobia*, fear*, underus*, underprescri*, underutiliz*, and barrier*, restricted to titles and abstracts on PubMed, Web of Science, Scopus, and Cochrane. A total of 362 records were retrieved, and 30 records were identified through backward citation searching. After screening, 53 studies were eligible for review. This review identified several barriers to CLZ use, which were grouped into three broad domains: clinician‐related, medication‐ and patient‐related, and administrative‐related barriers. Key barriers included difficulties in recognising Treatment‐Resistant Schizophrenia (TRS) and identifying candidates for CLZ, lack of training and confidence, negative attitudes toward CLZ, variability in treatment pathways, concerns about CLZ's adverse effects, and challenges in convincing carers of the need for CLZ, insufficient resources, unclear professional roles, and fragmentation of services. The vast majority of identified barriers stem from misconceptions or negative attitudes toward CLZ, which unjustifiably limit patient access to this effective treatment, and can be addressed through the implementation of existing facilitating strategies. In such contexts, the term clozaphobia seems appropriate and its use may help to raise awareness and promote critical reflexion on this subject. With the end of the clozapine risk evaluation and mitigation strategy, it is now time to push society to start producing higher dose pills and a long‐acting injectable version of clozapine.

## 1 Introduction

### 1.1 Schizophrenia

Schizophrenia is a complex psychiatric disorder that manifests not only, but particularly in late adolescence and young adulthood (Wilson et al. 2019; Wilson et al. 2019). This disorder impacts patients' quality of life, physical and mental health, socio‐economic status and mortality (Agid et al. 2024; Jakobsen et al. 2022), and is thought to affect between 0.45% and 1% of the world's population (Jakobsen et al. 2022; Rezaie et al. 2022). The relapse rate reaches 80% (Agid et al. 2024).

The longer the duration of untreated psychosis (DUP), the worse the prognosis (Cirulli 2005; Agid et al. 2024; Walker et al. 2024). Patients who are not adequately treated impose a high level of stress on society (Rezaie et al. 2022). In 2019, the economic burden of schizophrenia in the United States of America (USA), affecting around 3.9 million people, was estimated at 343.3 billion US dollars (Torrey and Lieberman 2024).

Pharmacotherapy with antipsychotics remains the intervention of choice, with psychosocial interventions being useful as adjuncts (Rubio and Kane 2019; Shangraw et al. 2024; Takeuchi et al. 2016; Waserman and Criollo 2000). Early response to treatment is a strong predictor of good clinical outcome (Agid et al. 2024). First‐line treatment usually consists of any third‐generation antipsychotic (TGAP) or second‐generation antipsychotic (SGAP) (Bogers et al. 2014; Wilson et al. 2019). Guidelines exist, but these tend to be country‐specific and are often missing a concise yet comprehensive algorithmic approach. Recently, authors from all around the world collaborated to develop a consensus guideline focused on the pharmacological treatment of schizophrenia: the International Guidelines for Algorithmic Treatment (INTEGRATE). Key recommendations included a focus on metabolic health from treatment initiation, timely assessment and management of non‐response, symptom domain‐specific interventions, mitigation of side‐effects, and the prompt use of clozapine (CLZ) in cases of treatment resistance (McCutcheon et al. 2025).

### 1.2 Treatment‐Resistant Schizophrenia

Treatment‐resistant schizophrenia (TRS) is a common condition. It is estimated that a fourth (Agid et al. 2024; Oloyede et al. 2023) or a third (Jakobsen et al. 2022) of patients with first psychotic episode will have TRS. When using broader definitions of TRS, the incidence can be as high as 30%–60% (Cirulli 2005; Mistry and Osborn 2011; Filia et al. 2012; Agid et al. 2024; Shangraw et al. 2024; Verdoux et al. 2025).

TRS is broadly defined as SCZ patients that have not responded adequately to two different APs. A lack of consensus defining TRS may explain inconsistent results (Mistry and Osborn 2011; Rubio and Kane 2019; Howes et al. 2021; Lappin et al. 2022).

The Treatment Response and Resistance in Psychosis (TRRIP) consensus established three key elements: a confirmed diagnosis of SCZ; adequate pharmacological treatment; and persistence of significant illness (Howes et al. 2016; Lappin et al. 2022; Rubio and Kane 2019). The clinical presentation of TRS varies and so specific symptomatic subdomains should be explicitly identified (Howes et al. 2016; Howes et al. 2021).

Some authors recommend that the two previous AP trials should include a first‐generation FGAP (Mistry and Osborn 2011), a SGAP (Agid et al. 2024; Leucht et al. 2008), or even consider CLZ as the second antipsychotic trial (Kahn et al. 2018).

Compared with non‐resistant SCZ, TRS is associated with worse severity, economic burden, comorbidity and psychosocial adjustment (Agid et al. 2024; Rezaie, Nazari, Safari‐Faramani, et al. 2022). The quality of life of patients is estimated to be one fifth lower. Annual direct medical costs of TRS in the USA are more than 34 billion US dollars and the costs in health resources are up to 10 times higher. Around 80% of the annual health costs associated with SCZ in the USA are attributable to TRS (Cotes et al. 2021; Howes et al. 2021).

‘Pseudo‐resistance’ can happen after misdiagnosis or inadequate treatment due to lack of adherence, doses of the medication, genetic variants, comorbidities, polypharmacy, adverse effects, pharmacodynamics and pharmacokinetics (Agid et al. 2024; Howes et al. 2021). It is important that patients who do not adequately respond to antipsychotics be reevaluated to exclude or address causes other than non‐responsiveness to medication, that is, the possibility of pseudo‐resistance. In particular, non‐adherence to oral antipsychotic treatment should be monitored to rule out pseudo‐resistant cases of TRS. Moreover, patients with TRS who take their medication as required may have subtherapeutic antipsychotic plasma levels, secondary to pharmacokinetic factors (H. Kim and Lee 2020). Another overlooked, underestimated cause of pseudo‐TRS is ‘pseudo‐schizophrenia’: patients suffering from organic psychosis that do not respond properly to the treatment because they were misdiagnosed as having schizophrenia (Gama Marques 2021, 2022; Gama Marques and Finsterer 2025; Gama‐Marques et al. 2025). Indeed, many patients can suffer with an important delay before the correct diagnosis, theragnosis and prognosis is reached (Gama Marques and Bento 2020).

### 1.3 Clozapine

CLZ, the first second‐generation antipsychotic, was synthesised in 1956 and began to be used in Europe in 1971 (Cetin 2014). It was approved in the USA for the treatment of TRS in 1989 (Rubio and Kane 2019). To date, CLZ is the only first‐line evidence‐based drug recommended for TRS by the Food and Drug Administration and the European Medicines Agency, among others organisations. These indications include all cases of TRS (Carroll et al. 2024).

CLZ is also recommended for suicidal or violent behaviour, dual diagnosis, tardive dyskinesia, psychogenic polydipsia, treatment‐resistant bipolar affective disorder, psychoses associated with post‐traumatic stress disorder and Parkinson's syndrome (Blagden et al. 2020; D. L. Kelly et al. 2017; D. L. Kelly and Love 2019; Moody and Eatmon 2019; Nielsen et al. 2009; Rubio and Kane 2019; Singh et al. 2019; Martinotti et al. 2022). Its use is usually limited due to its adverse effects (Grover, Balachander, et al. 2015). CLZ is only licensed for TRS patients aged 16 and over (Cirulli 2005) but it should be considered for all ages (Walker et al. 2024).

The superiority of CLZ over other antipsychotics has been replicated in several systematic reviews and meta‐analyses. CLZ is superior as a first‐ and second‐line therapy in TRS (Okhuijsen‐Pfeifer et al. 2018). Response rates in TRS are of 40% (Lappin et al. 2022), 30%–60% (Filia et al. 2012; Nielsen et al. 2009), 60% (Mistry and Osborn 2011), 50%–80% (Grover, Hazari, et al. 2015) or 80% (Jakobsen et al. 2022; Walker et al. 2024). CLZ should always be trialled in patients with TRS (Swinton and Ahmed 1999).

CLZ is effective in improving psychotic symptoms, sexual dysfunction, tardive dyskinesia, dual diagnosis conditions, aggressive and self‐harming behaviour (Agid et al. 2024; Torrey and Lieberman 2024; Verdoux et al. 2025). Initial trials demonstrated superiority in patients with prominent positive symptoms and negative symptoms in TRS (Agid et al. 2024; Grover, Balachander, et al. 2015; Grau‐López et al. 2020; Verma et al. 2020), improving patients' psychosocial functioning (Agid et al. 2024). Patients with good adherence to CLZ experience fewer relapses, hospitalisations, appointments, polypharmacy, leading to less healthcare costs (Agid et al. 2024; D. L. Kelly et al. 2017; D. L. Kelly and Love 2019; Lappin et al. 2022; Mistry and Osborn 2011). CLZ overall mortality is around half compared with other antipsychotics (Rubio and Kane 2019; Agid et al. 2024; Wilson et al. 2019).

Still the classic superiority of CLZ has been challenged by recent meta‐analyses (Schneider‐Thoma et al. 2025; Kotochinsky et al. 2026), with other authors leaving some room for doubt (Andrade 2025). Still the discussion is far from closed (Erlianti et al. 2025) and the most critical voices argue that there is no need for more research, but for more honesty when psychiatrists publish unwelcome research (Gøtzsche 2025).

### 1.4 Underprescription of Clozapine

The available data suggest significant variability between regions and clinical settings, with many healthcare professionals failing to initiate CLZ (Agid et al. 2024; Tungaraza and Farooq 2015; Verdoux et al. 2025). Finland has the highest prescription rate (189.2 per 100,000 inhabitants), while Japan has the lowest (0.6 per 100,000 inhabitants), with most countries reporting rates of less than 100 per 100,000 inhabitants (Agid et al. 2024; Rubio and Kane 2019). In China, CLZ is prescribed to around 60% of individuals with SCZ (Torrey and Lieberman 2024; Wilson et al. 2019). The use of CLZ has the potential to be improved (Agid et al. 2024). The estimated rate of use among all eligible patients ranges from 14% to 50% (Farhadian et al. 2011), and 44%–52% of outpatients with an indication for CLZ remain CLZ‐*naïve* (Jakobsen et al. 2023).

The delay in CLZ initiation varies significantly: from 1 to 10 years after the diagnosis of TRS (Daod et al. 2019; Gee et al. 2013; Grant et al. 2024; Grover, Hazari, et al. 2015; Jakobsen et al. 2023; Mistry and Osborn 2011; Rezaie et al. 2022; Tungaraza and Farooq 2015). This delay is due to clinicians' preference for multiple trials, high doses and polypharmacy with others APs (Daod et al. 2019; Grover, Balachander, et al. 2015; Grover, Hazari, et al. 2015; Jakobsen et al. 2022, 2023; Lappin et al. 2022; Mistry and Osborn 2011; Rezaie, Nazari, Safari‐Faramani, et al. 2022; Singh et al. 2019). Approximately 20% of all patients with SCZ and up to 65% of those taking CLZ are subject to antipsychotic polypharmacy (Jakobsen et al. 2022; D. L. Kelly and Love 2019; Rubio and Kane 2019; Tang et al. 2017) even without evidence supporting superiority over CLZ monotherapy (Mistry and Osborn 2011).

Clozapine is known to cause agranulocytosis, who frightened physicians, families and patients for decades. But not all cases of agranulocytosis occurring in people taking clozapine are caused by clozapine. The widely used threshold criterion‐based diagnosis overestimates the risk of agranulocytosis, despising other possible causes like benign ethnic neutropenia that shall always be ruled out (Taylor et al. 2024). Indeed, a meta‐analysis suggested a lower incidence of agranulocytosis than previously estimated in patients taking clozapine, demanding a revision of the approach that both psychiatrists and supervising organisations often take when talking about clozapine (Magistri and Mellini 2023).

Success of CLZ is strongly dependent on the timing of its introduction (Jakobsen et al. 2022). The greater the number of previous antipsychotics trials and the number of previous hospitalisations, the lower the likelihood of an effective response to CLZ (Agid et al. 2024; Bogers et al. 2014; Cirulli 2005; Grant et al. 2024; Jakobsen et al. 2022, 2023; Oloyede et al. 2023). Schneider‐Thoma et al. (2025) point out that patients with a shorter DUP could have a better prognosis. Delaying CLZ treatment in TRS is associated with worse levels of clinical care, functionality, quality of life, burden on society, prognosis, morbidity, mortality and healthcare costs (Jakobsen et al. 2022; D. L. Kelly et al. 2017; Oloyede et al. 2023; Rezaie, Nazari, Safari‐Faramani, et al. 2022; Rubio and Kane 2019).

Some serious trouble, but not all, could be avoided if CLZ was started earlier in the life of the most severely psychotic patients. It is widely known that homeless psychotic patients are less likely to receive CLZ (Shangraw et al. 2024). Up to 30% of episodes of mass violence are attributed to men with untreated psychoses (Torrey and Lieberman 2024), who sooner or later will end incarcerated or on the streets. An earlier use of CLZ could be part of a broader strategy in order to avoided, at least, some of the fatal cases (both suicide or homicide) secondary to psychosis relapse.

### 1.5 Clozaphobia

It is crucial to understand the barriers that contribute to the underuse of CLZ, because patients have better outcomes when using clozapine for the treatment of TRS. In this article, we would like to adopt the term ‘clozaphobia’, an expression used by a couple of authors with similar definitions, at least, in the last 10 years: the fear of prescribers of clozapine for treatment of schizophrenia (Cetin 2014); the fear of clozapine's adverse effects (Sreeraj et al. 2017); and the fear of prescribing clozapine (Pandarakalam 2019).

Another example of an extreme fear of a highly effective medication is the fear regarding the prescription of chloramphenicol. This fear is quite irrational as general practitioners should expect to see aplasia secondary to chloramphenicol in one patient every one hundred years (Cox and Roderick 1995). Indeed, chloramphenicol's activity outlines its contraindications as it is a valuable agent in the treatment of severe life‐threatening conditions, such as those caused by *Salmonella typhi* or *Haemophilus influenza* (Oong and Tadi 2023). Despite its underuse for over a half a century, chloramphenicol remains an effective antibiotic when used appropriately, and the continued increase in antimicrobial resistance to currently available antibiotics may necessitate the increased use of chloramphenicol in the future (Wiest et al. 2012).

Reasons for avoidance of CLZ are rarely recorded in clinical files (Cotes et al. 2021; Jakobsen et al. 2022, 2023; Oloyede et al. 2023). Knowledge, beliefs, attitudes and prior experiences of patients, carers and healthcare professionals play an important role in CLZ underuse (Carroll et al. 2024; Ristic et al. 2021). These subjective experiences influence prescription rates, adherence to treatment, therapeutic results and quality of life (J. Kim et al. 2005; Sloan et al. 1997; Waserman and Criollo 2000). It is crucial to understand the barriers contributing to the underuse of CLZ (Grant et al. 2024; Rezaie et al. 2022; Jakobsen et al. 2022).

Factors contributing to the delay in CLZ initiation can be divided into three domains: clinician‐related barriers, medication‐ and patient‐related barriers and administrative or institutional barriers (Farooq et al. 2019). The main objective of this review is to contribute for a better understanding and management of clozaphobia.

## 2 Methods

This narrative review followed the Preferred Reporting Items for Systematic Reviews and Meta‐Analyses (PRISMA) guidelines. Studies that could not be accessed, as well as letters to the editor, editorials, conference abstracts, commentaries, and animal studies were excluded. Only English articles were included.

**FIGURE 1:**
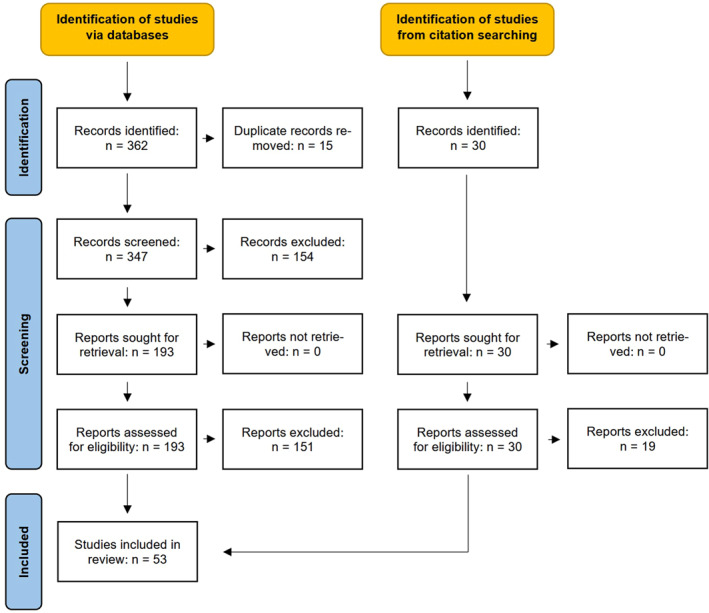
The PRISMA flow diagram for clozaphobia literature review.

In May 2025, a literature search was conducted using the databases PubMed, Web of Science, Scopus, and Cochrane. The search strategy used the following terms, combined with the Boolean operators OR and AND, and limited to the title and abstract fields: ((fear* OR underus* OR underprescri* OR underutiliz* OR barrier*) AND *clozapine*) OR *clozaphobia*. The Mendeley Reference Manager software was used to import and store the total 362 records retrieved, and 15 duplicates were removed. After screening of abstracts 154 records were excluded. A total of 193 records were eligible for full‐text screening, and an additional 30 records were identified through backwards citation searching. In total, 53 studies were included in the review (Figure 1). The data are available on request from the corresponding author.

## 3 Results

### 3.1 Clinician‐Related Barriers

Clinicians are in a privileged position to identify who has an indication for using CLZ, to make the decision to start therapy, to monitor patients and to positively influence their attitudes (Agid et al. 2024; Oloyede et al. 2023; Rezaie et al. 2022; Rezaie et al. 2022b). Globally, there has been a decline in the number of psychiatrists working in the public service, especially in regions with limited resources. This raises the possibility that other professionals (e.g., advance‐practice nurse practitioners, psychiatric‐trained physician assistants and board‐certified psychiatric pharmacists) could contribute to improving access to psychiatric care (D. L. Kelly and Love 2019). For this reason, the attitudes of other healthcare professionals with an increasing role in prescribing CLZ are also included here (Okhuijsen‐Pfeifer et al. 2019).

In general, clinician‐related barriers include: lack of knowledge, training, experience and confidence in prescribing CLZ; difficulties in initiating and maintaining therapy; concerns about risk and adverse effect assessment and mitigation strategies; doubts about patient adherence; and difficulties in properly identifying cases of TRS. In Oloyede et al. (2023), clinician‐related barriers were the third most frequently mentioned theme by participants (20%, *n* = 17) and included subthemes such as clinician attitudes, formulation, prescriber confidence, side‐effects, treatment pathways and TRS identification. Below, we organise and explore barriers in more detail, using an adapted version of this subdivision.

#### 3.1.1 TRS Formulation and Identification

The correct identification of cases of TRS is an area where clinicians feel poorly trained (Walker et al. 2024). Many believe that TRS is a rare condition, limited to a small percentage of individuals with SCZ, or that it only manifests itself in the early stages of the disorder and cannot appear later. Furthermore, there is a belief that it is necessary to establish chronicity in order to formulate a proper diagnosis of TRS (Agid et al. 2024).

Some clinicians also report difficulties in distinguishing between cases of total and partial resistance to treatment, which is influenced by the limited use of objective scales (e.g., Positive and Negative Syndrome Scale). In some cases, there may be an erroneous identification of pseudo‐resistance as TRS (Agid et al. 2024; Oloyede et al. 2023). In Rezaie, Nazari, Safari‐Faramani, et al. (2022) and Tungaraza and Farooq (2015), 60% (*n* = 169) and 36.2% of psychiatrists (*n* = 88), respectively, found it difficult to quickly identify which patients would benefit from a transition to CLZ treatment. Cirulli (2005) observed that 29% of psychiatrists (*n* = 17) refused to prescribe CLZ, with 12% (*n* = 7) being due to a lack of cases considered suitable.

However, in Grant et al. (2024), 79% (*n* = 57) and 88% of clinicians (*n* = 63) said that difficulties in identifying eligible patients or diagnostic uncertainties, respectively, were not factors delaying CLZ prescription.

#### 3.1.2 Indications for Clozapine use

Patients with psychotic disorders often have a history of suicidal ideation or substance use disorder. In Verma et al. (2020), 13.5% of patients (*n* = 7) had tobacco dependence, 28.8% (*n* = 15) had already manifested suicidal behaviour, and 23.1% (*n* = 12) had tried suicide attempts. According to Grant et al. (2024), 78% of clinicians (*n* = 52) recognised that CLZ reduces the risk of suicide and is associated with lower mortality compared to other APs. In Rezaie, Nazari, Safari‐Faramani, et al. (2022), 70% of clinicians (*n* = 197) recognised CLZ's effect in reducing suicide risk, in Singh et al. (2019), 84% (*n* = 138) recognised its usefulness in recurrent suicidal behaviour and, in Tungaraza and Farooq (2015), only 18.9% (*n* = 46) did not know that this medication reduces the risk of suicide. However, in Nielsen et al. (2009), only 3% (*n* = 3) stated that they would use CLZ in cases of suicidality and in Cirulli (2005) only 29% (*n* = 10) considered that CLZ contributed to a reduction in aggression, and only 18% (*n* = 6) agreed with its action in indirectly reducing mortality.

This lack of knowledge is also reflected in the clinical approach to DD cases. In Rezaie, Nazari, Safari‐Faramani, et al. (2022), 45% of clinicians (*n* = 127) believed that CLZ was associated with a reduction in substance use, and, in Grau‐López et al. (2020), only 30.8% (*n* = 61) reported considering its use in those contexts, despite 59.9% (*n* = 119) knowing it is a recommended medication. Similarly, in Tungaraza and Farooq (2015), 42.7% of psychiatrists (*n* = 104) did not know about the evidence on CLZ's reduction of alcohol and drug consumption in patients with SCZ. In Shangraw et al. (2024), no relationship was found between the start of CLZ therapy and previous suicide attempts, violent behaviour or substance abuse, which may suggest a lack of knowledge of these indications among prescribers. In Filia et al. (2012), having ongoing case management needs (e.g., a history of SUD) was considered a barrier to the transition of patients from public community mental health services to general practitioner (GP)‐shared or private care, scoring between 2.1 and 2.8 (3 being ‘very important’), respectively.

Regarding other CLZ indications, in Singh et al. (2019), 76% of residents (*n* = 125) were aware of its use in TD or intractable extrapyramidal symptoms (EPSs), and 56% (*n* = 92) in Parkinson's‐related psychosis; in Nielsen et al. (2009), 19% of psychiatrists (*n* = 19) mentioned its use in SZA, 17% (*n* = 17) in BAD, and 4% (*n* = 4) in TD; and in Tungaraza and Farooq (2015), 45.4% of psychiatrists (*n* = 104) reported prescribing CLZ for conditions other than TRS, namely resistant SZA (52 mentions), resistant BAD (38 mentions), borderline personality disorder (25 mentions), SCZ with intolerance to other APs (13 mentions), and less frequently for Parkinson's‐related psychosis, persistent delusional disorder, psychotic depression and severe head injury (each with less than 10 mentions).

There is often the idea that young or advanced age and co‐morbid medical conditions limit treatment with CLZ (Agid et al. 2024). Professionals often fear that adverse effects might not be reversible in the young (Oloyede et al. 2023). In Grover, Balachander, et al. (2015), 51.3% of psychiatrists (*n* = 281) also feared the medical complications associated with CLZ. In Verma et al. (2020), 26.9% of patients (*n* = 14) had physical and 46.2% (*n* = 24) had psychiatric comorbidities. Excluding patients with comorbidities from CLZ treatment means excluding a large proportion of its potential candidates.

In Tungaraza and Farooq (2015), 90% of psychiatrists (*n* = 219) agreed that the use of CLZ was appropriate in the young. Yet, in Grover, Balachander, et al. (2015), 24.3% (*n* = 133) were hesitant to prescribe CLZ to patients of paediatric age, and, in Cirulli (2005), 17.2% (*n* = 10) working in adolescent units did not prescribe CLZ due to the patients' age, with 8.6% (*n* = 5) only prescribing it to patients aged 16 or over. In Walker et al. (2024), 33.5% (*n* = 12) highlighted the lack of formal training on CLZ's use in adolescents.

In Grover, Balachander, et al. (2015), 32.5% of psychiatrists (*n* = 178) were also hesitant to prescribe CLZ to patients of older age. In Davis et al. (2013), CLZ initiation at an older age was associated with higher rates of all‐cause and non‐patient‐related discontinuations, but was not associated with patient‐related discontinuations.

#### 3.1.3 Clinician Training and Confidence

In Walker et al. (2024), 32.4% of clinicians (*n* = 11) reported having prescribed CLZ in the previous 5 years, while 44.1% (*n* = 15) had never done so. In Singh et al. (2019), 63% (*n* = 103) had patients with indication, but only 68% of those (*n* = 70) reported having between 1 and 5 patients undergoing CLZ treatment. In Gee et al. (2013), 56% (*n* = 79) said they had participated in CLZ initiation in the previous 6 months, while 14% (*n* = 20) had never done so. In Farhadian et al. (2011), 52.0% (*n* = 61) had not prescribed CLZ for more than a year, although 55.0% (*n* = 65) claimed to follow the guidelines.

In Okhuijsen‐Pfeifer et al. (2019), psychiatrists followed‐up an average of 97.4 patients with SCZ spectrum disorders, of whom 2.7 had an indication for CLZ use but were not taking it. Nurse practitioners followed up an average of 76.2 patients and reported 7.9 in the same condition. Daod et al. (2019) found that, among patients with a formal indication for CLZ use, only 42% were actually receiving it. In Grover et al. (Grover, Balachander, et al. 2015; Grover, Hazari, et al. 2015), 28.46% of patients previously followed up by other psychiatrists were not taking CLZ despite having indication for it. In Gee et al. (2013), more than 35% of clinicians (*n* = 50) estimated that up to 20% of their CLZ eligible patients were not taking it. These data reinforce the widespread perception that CLZ is still underused.

However, some studies show a more proactive tendency to CLZ prescription. Grant et al. (2024) reported that 92% of clinicians (*n* = 54) had 10 or more patients being treated with CLZ, and only 3% (*n* = 2) had never prescribed it. In Rezaie, Nazari, Safari‐Faramani, et al. (2022), 69% (*n* = 195) reported having five or more patients taking CLZ, and 93% (*n* = 262) had prescribed it at some point in their careers. In Ristic et al. 2021, 60% (*n* = 97) indicated they had already treated at least 10 patients with CLZ, and only 2.5% (*n* = 4) had no patients under this treatment at that time. In Tungaraza and Farooq (2015), 65.2% (*n* = 144) had started CLZ treatment in the previous 6 months. Finally, in Nielsen et al. (2009), psychiatrists had an average of 8.9 patients on CLZ, with 19.0% (*n* = 19) having prescribed it for the last time in the previous month, 12.0% (*n* = 12) in the previous 3 months, 21.0% (*n* = 21) in the previous 6 months, 9.0% (*n* = 9) in the previous year, 32.0% (*n* = 32) more than a year ago, and 7.0% (*n* = 7) had never prescribed it.

#### 3.1.4 Years of Experience and Prescription Rate

In Rezaie, Nazari, Safari‐Faramani, et al. (2022), there was a correlation between psychiatrists with more than 15 years' experience and a higher rate of CLZ prescription. These psychiatrists also believed that CLZ was more effective, that its initiation was safe, and that the treatment was related to a reduction in substance abuse and risk of suicide. They also reported good clinical experience in managing CLZ and its adverse effects and the perception that CLZ was not being overprescribed. There was also a correlation between psychiatrists with less than 15 years' experience and the beliefs that it was difficult to initiate CLZ therapy and convince patients and carers about its initiation, greater fear of fatal AEs, the belief that CLZ had a greater likelihood of interactions with other medications and risk of death compared to other antipsychotics, lack of direct experience of CLZ's superiority, and the opinion that CLZ should be delayed, even in the absence of suitable alternatives. However, similar correlations do not appear in all studies. In Daod et al. (2019), specialists were more familiar with the TRS’ guidelines compared to residents. Even so, 63% of residents (*n* = 79) supported initiating treatment according to the guidelines and only 25% (*n* = 32) said they would only start CLZ after three failed trials with other APs versus 47.6% (*n* = 83) and 39.5% of specialists (*n* = 69), respectively. Familiarity with the CLZ guidelines did not differ between more or less experienced specialists. These data suggest an inverse trend to that reported by Rezaie, Nazari, Safari‐Faramani, et al. (2022).

In Takeuchi et al. (2016), 54.8% of clinicians (*n* = 57) had never prescribed CLZ and had an average of 13.7 ± 21.3 years of work experience. In contrast, the 45.2% (*n* = 47) who had already prescribed it had an average of 9.9 ± 6.9 years' experience. In Tungaraza and Farooq (2015), 41.7% (*n* = 80) had fewer than five patients under CLZ therapy, despite having been working in their service for at least 7 years. In Nielsen et al. (2009), 7% of psychiatrists (*n* = 7) had never made the decision to initiate CLZ (although five of them were treating patients with this medication): all had more than five years' experience, were aged between 41 and 53, five were consultant psychiatrists, and all shared a more negative view of CLZ's efficacy and safety.

Clinicians who rarely prescribe CLZ, or who are considered inactive prescribers (i.e., those who have not prescribed CLZ in the previous month), tend to be less comfortable with its use (Cotes et al. 2021; Sharma et al. 2021). In Cotes et al. (2021), only 48% of inactive prescribers (*n* = 41) felt comfortable prescribing CLZ, compared to 95% of active prescribers (*n* = 55). Tang et al. (2017) found that prescribers classified as high‐volume (50.3%, *n* = 327), compared to low‐volume (49.7%, *n* = 323), were more likely to use CLZ [77% (*n* = 252) versus 43% (*n* = 139)], but also APP [91% (*n* = 298) versus 25% (*n* = 81)].

Clinicians with no experience in prescribing CLZ show greater hesitation about its efficacy and safety, a tendency to overestimate the barriers associated with its prescription and medication‐related mortality (Cotes et al. 2021; Sharma et al. 2021), and are more likely to deliberately delay its introduction (Jakobsen et al. 2023). In Takeuchi et al. (2016), among clinicians with prescribing experience, 77.2% (*n* = 44) considered CLZ to be effective, 38.6% (*n* = 22) believed it to be safe, 43.9% (*n* = 22) thought their patients were satisfied with the treatment, and 82.4% (*n* = 47) saw it as a necessary medication. Among clinicians with no prescribing experience, 70.2% (*n* = 33) thought it was effective, 21.3% (*n* = 10) that it was safe, 19.1% (*n* = 9) that their patients were satisfied with the treatment, and 74.4% (*n* = 35) that it was a necessary medication.

On the other hand, there are studies in which the correlation between experience and rate of CLZ prescription is not so clear. Grover, Balachander, et al. (2015) found a correlation between more years of clinical practice and a preference for combining CLZ with other antipsychotics, less appreciation of CLZ for managing psychotic disorders in general, more appreciation for managing negative symptoms, and a greater number of patients treated with it. In Ristic et al. 2021, no correlations were found between the number of patients under CLZ treatment, the years of professional experience and practice in a university setting. Singh et al. (2019) also found no differences in levels of comfort with prescribing between junior and senior residents. Moody and Eatmon (2019) identified no significant differences in CLZ prescription rates based on institutional affiliation, time spent in direct contact with patients or geographical location.

#### 3.1.5 Training and Knowledge

Generally, prescribers who have no specific experience with CLZ feel a lot of anxiety with its use (Oloyede et al. 2023). In Singh et al. (2019), only 18% of residents (*n* = 30) felt comfortable with prescribing, while 41% (*n* = 67) felt somewhat comfortable. In Moody and Eatmon (2019), clinicians who were more familiar and comfortable with the monitoring requirements were more likely to prescribe.

Experienced clinicians have noted that some younger staff members are unaware of key aspects of CLZ prescription they feel are not their responsibility (e.g., cardiometabolic monitoring), which leads them to conclude that residency training on CLZ is failing and that investment should be made in this direction (Blagden et al. 2020). Limited experience and inadequate training in the use of CLZ are barriers to prescribing according to 38% of clinicians (*n* = 62) in Singh et al. (2019), 48.4% (*n* = 47) in Moody and Eatmon (2019), 45% (*n* = 5) in Maryan et al. (2019), 71% (*n* = 173) in Tungaraza and Farooq (2015) and 12.0% (*n* = 7) in Cirulli (2005).

Professionals with less experience or knowledge about CLZ (e.g., due to insufficient knowledge or exposure during residency, frequent job changes) tend to overestimate AEs and not properly value the superior CLZ efficacy in TRS (Tungaraza and Farooq 2015). In addition, limited experience is known to contribute to non‐evidence‐based choices, such as APP, especially if CLZ is not commonly used in the professionals' clinical setting (Rubio and Kane 2019).

In Singh et al. (2019), 66.7% of residents (*n* = 109) did not have specific training on CLZ during residency nor did they attend a CLZ‐dedicated clinic and 83% (*n* = 136) felt the need for more training and updates on it, stating that this would make them feel more comfortable when prescribing (Rezaie, Nazari, Safari‐Faramani, et al. 2022). In Grau‐López et al. (2020), 61.8% of clinicians (*n* = 123) considered that managing CLZ treatment is more difficult than with other APs. Of these, 35.6% (*n* = 44) attributed this difficulty to the lack of training, with 20% (*n* = 40) stating that they had not received any formal training during residency. In Cotes et al. (2021), only 22% (*n* = 31) had formal training on CLZ. In Singh et al. (2019), 46% of residents who did not attend a CLZ clinic (*n* = 52) felt inadequately trained versus 20% of residents who did (*n* = 10). However, no significant differences were found between the two groups in regards to knowledge about CLZ's indications. In Walker et al. (2024) and Grau‐López et al. (2020), 58.8% (*n* = 19) and 27.8% of clinicians (*n* = 55), respectively, pointed out facilitators such as practical CLZ training through observation of experienced colleagues and dedicated time to managing CLZ cases. In Moody and Eatmon (2019) and Maryan et al. (2019), 59.8% (*n* = 56) and 72% of clinicians (*n* = 8), respectively, also mentioned in‐service educational sessions.

The lack of training is not only mentioned by prescribers, but also by pharmacists about CLZ dispensing, which is considered to influence patient's adherence (Ismail et al. 2018). In Wilson et al. (2019), 60.3% of pharmacists (*n* = 73) had received training on CLZ prior to the implementation of the community pharmacy CLZ‐dispensing service. Of the 39.7% (*n* = 48) who had not received training, 47.9% (*n* = 23) did not consider it necessary. Even so, confidence and knowledge about safe CLZ dispensing was identified as a main facilitator. Of the untrained pharmacists, 45.8% (*n* = 22) wanted access to training (e.g., community pharmacies with a CLZ service, information on different brands and strategies to ensure a safe and timely supply).

However, in Grant et al. (2024), the majority of clinicians did not consider lack of training and confidence to be factors that delayed CLZ initiation. In Oloyede et al. (2023), only 14% of clinicians (*n* = 16) emphasised the importance of investing in prescriber training and only 8% (*n* = 9) mentioned the need for more guidance. In Grover, Balachander, et al. (2015), only 0.2% of psychiatrists (*n* = 1) said they would refer a patient to another specialist if they were reluctant to prescribe, while 46.0% (*n* = 252) would never do so. In Tungaraza and Farooq (2015), 75.4% (*n* = 169) claimed to have had good exposure to CLZ during their residency. In Rezaie, Nazari, Safari‐Faramani, et al. (2022), more than 80% (*n* = 226) reported having read articles on CLZ during their careers and 97% (*n* = 274) said they had access to counselling services if they had any particular concern. Some clinicians also reported receiving updates on recent studies via leaflets, in‐service training or team meetings and pointed out having access to training through professional organisations such as the American Psychiatric Association (Carroll et al. 2024). Therefore, opinions are divided on the importance of greater access to CLZ guidelines: in Grant et al. (2024), 70% of clinicians (*n* = 45) considered it a relevant facilitator versus only 8.8% of clinicians (*n* = 3) in Walker et al. (2024). Curiously, sometimes there are no clear differences between professionals who have or have not received formal training on CLZ during residency, particularly regarding their prescribing rates in the previous month, perceptions of safety, efficacy and comfort or knowledge about this medication (Cotes et al. 2021). It has also been suggested prescribing may be influenced by the prescriber's personality traits (e.g., high levels of danger avoidance leading to reluctance in CLZ prescribing) (Rezaie et al. 2022).

#### 3.1.6 Lack of Confidence About Initiation and Monitoring

In Rezaie, Nazari, Safari‐Faramani, et al. (2022), 81.2% of psychiatrists (*n* = 229) mentioned their lack of personal experience in initiating and monitoring CLZ therapy as a barrier to its use. Specifically regarding CLZ initiation, there are varying opinions on whether it can be carried out in a community setting, or whether hospitalisation is necessary. In Grant et al. (2024), 51% of clinicians (*n* = 39) considered that this factor contributes, at least somewhat frequently, to delays in CLZ initiation, although 46% (*n* = 35) stated that they would not consider it an impeding factor. Some clinicians admit that CLZ titration can be challenging, as it requires frequent dose increases or adjustments, depending on the patients' metabolic phenotype, the presence of metabolic inhibitors or inducers, and the need to monitor and manage early AEs. Clinicians also have doubts concerning the CLZ initiation in acute and agitated patients, where a faster course of action may be required, which could theoretically be incompatible with a slow and safe titration (Agid et al. 2024). However, in Daod et al. (2019) and Gee et al. (2013), 69.5% (*n* = 205) and 48% of clinicians (*n* = 69) claimed to be familiar with CLZ initiation, respectively.

Specifically regarding CLZ monitoring, in Grau‐López et al. (2020), 61.8% of clinicians (*n* = 123) considered the CLZ to be more difficult to manage than other APs and, of these, 57.1% (*n* = 70) referred the difficulty of pharmacovigilance and haematological monitoring. In Ismail et al. (2018), clinicians were aware of the requirements for CLZ prescribing and the services available in their healthcare system for haematological monitoring. Despite such services being readily available, the lack of a national‐wide program for haematological monitoring and performance measurement was said to difficult the assessment of CLZ's safety and efficacy (Carroll et al. 2024).

Guidelines recommend the use of therapeutic drug monitoring (TDM) (i.e., the measurement of CLZ plasma concentrations) during CLZ therapy. In Ristic et al. 2021 and Daod et al. (2019), 82.0% (*n* = 132) and 62% of clinicians (*n* = 183) considered it a useful measure and, in Nielsen et al. (2009), 90.0% (*n* = 89) performed it. However, only 59.1% of clinicians (*n* = 118) in Grau‐López et al. (2020) and 29% (*n* = 10) in Cirulli (2005) would routinely perform TDM and only 15% (*n* = 44) in Daod et al. (2019) knew the recommended therapeutic plasma concentrations. Some clinicians report the unavailability of TDM in their clinical setting or delays in TDM results and believe that these factors make optimal CLZ titration impossible (Agid et al. 2024).

#### 3.1.7 Perceptions About Clozapine Efficacy and Prescription Rates

In Grant et al. (2024), 92% of clinicians (*n* = 54) consider pharmacological treatment to be the most important treatment modality in SCZ, which also received an average score of 8.4 (10 being ‘the most important’) in Nielsen et al. (2009). Most clinicians consider CLZ to be an effective antipsychotic. In Rezaie, Nazari, Safari‐Faramani, et al. (2022) and Grover et al. (Grover, Balachander, et al. 2015; Grover, Hazari, et al. 2015), 74% (*n* = 209) and 95.6% of clinicians (*n* = 524), respectively, considered CLZ to be more effective than other APs. In the latter study, psychiatrists gave CLZ an average score of 7.53 (on a scale of 0–10) for its overall effectiveness in psychotic disorders, and an average score of 5.47 for its effectiveness in the negative symptoms of SCZ. In Takeuchi et al. (2016), 77.2% (*n* = 44) considered CLZ to be effective or extremely effective. In Gee et al. (2013), clinicians rated CLZ with 8 points (10 being ‘much more effective’), when comparing it with other antipsychotics in the treatment of SCZ. In Nielsen et al. (2009), the average rating regarding its efficacy and safety was 8.0. In Daod et al. (2019), 90.6% of clinicians (*n* = 267) believed in its efficacy for TRS, and 66.7% (*n* = 197) for treatment‐responsive SCZ.

In Verma et al. (2020), patients on CLZ treatment in the previous 3 months had a mean total score of 52.25 on the Positive and Negative Symptoms Scale (i.e., indicating very mild or mild symptomatology), suggesting good symptomatic response. All showed an improvement in global functioning in the Clinical Global Impression Severity of Symptoms (CGISS) scale: 69.2% (*n* = 36) were ‘much improved’ and 30.8% (*n* = 16) were ‘minimally improved’. Moreover, 53.8% (*n* = 28) achieved clinical remission according to criteria: a score of 3 or less on items P1, P2, P3, N1, N4, N6, G5 and G9 of the PANSS (symptomatic criterion), maintained for a minimum of six consecutive months (temporal criterion). In Sloan et al. (1997), patients' initial average score on the Brief Psychiatric Rating Scale (BPRS) was 65, which decreased to 46 after six months of CLZ therapy (i.e., showing clinical improvement), with increased patient satisfaction. In Davis et al. (2013), the average final BPRS score under CLZ therapy was 39, while the average score after discontinuing CLZ and taking a stable dose (≥ 3 months) of another AP was 52, revealing a statistically significant increase in patients' psychopathology. However, in Rezaie, Nazari, Safari‐Faramani, et al. (2022), 83% of psychiatrists (*n* = 234)—especially those without academic position—had never experienced the superiority of CLZ first‐hand.

Regarding the perception of CLZ's prescription rates, in Oloyede et al. (2023), 38% of clinicians (*n* = 47) considered that CLZ is adequately prescribed, 37% (*n* = 46) believed that it is under‐prescribed, 23% (*n* = 28) had no definite opinion, and only 2.3% (*n* = 3) thought that it is over‐prescribed. In Tungaraza and Farooq (2015), 80.7% (*n* = 184) agreed that there is a delay in the introduction of CLZ.

#### 3.1.8 Knowledge About and Prescription According to Guidelines

In Daod et al. (2019), 66% of clinicians (*n* = 195) reported being familiar with SCZ guidelines and 64.4% (*n* = 190) were familiar with CLZ guidelines. In Okhuijsen‐Pfeifer et al. (2019), 85% of psychiatrists (*n* = 95) and 90% of nurse practitioners (*n* = 37) and, in Gee et al. (2013), 81% of clinicians (*n* = 113) were at least moderately familiar with them. In Grover, Balachander, et al. (2015), 61.5% (*n* = 337) rated their knowledge on CLZ as ‘good’ and 34.5% (*n* = 189) as ‘very good’. In Ismail et al. (2018), clinicians were able to name the different local CLZ protocols regarding dosing, AE's management, dispensing and rechallenge strategies. They were familiar with at least one international SCZ treatment guideline, usually using the ones they were most familiar with (i.e., the ones taught during their residency or adopted in their clinical setting). They were also aware that APP is not recommended for the treatment of TRS before CLZ has been previously trialled. In Singh et al. (2019), 99% of clinicians (*n* = 162) were aware of CLZ's use in TRS and, in Nielsen et al. (2009), 87.8% (*n* = 86) knew that CLZ should be started after two unsuccessful antipsychotic trials. In Moody and Eatmon (2019), clinicians scored an average of 85.6% on the knowledge about CLZ. However, no statistically significant differences were found in the prescribing rate between clinicians with scores of 60% or less and those with scores of 80% or more.

In Swinton and Ahmed (1999), in 28% of TRS cases (*n* = 9), psychiatrists stated that treatment with CLZ was not started because patients had no indication for its use. This study seems to be an exception to what has been observed in the vast majority of other studies on the knowledge of CLZ guidelines.

In Nielsen et al. (2009), 99% of psychiatrists (*n* = 99) stated that they would use CLZ in TRS. Nevertheless, many clinicians deliberately delay prescribing CLZ despite being familiar with the guidelines (Ismail et al. 2018). In Verma et al. (2020), before they started CLZ, all patients had been diagnosed with SCZ for an average of 10.73 years, had been under psychopharmacological treatment for 25.9 months (with an average of 2.28 different medications), 42.3% (*n* = 22) had been hospitalised before and all had a continuous course of the disorder for at least 2 years. In Grau‐López et al. (2020), 54.5% of clinicians (*n* = 108) indicated that they would only consider introducing CLZ between three and 5 years after the diagnosis of SCZ, and more than half would only use it after at least two AP trials.

According to 59.7% of clinicians (*n* = 58) in Moody and Eatmon (2019), one of the main barriers for CLZ use is the preference for other pharmacological strategies (i.e., multiple trials and excessive switching, high doses of other antipsychotics, long acting‐injectables or antipsychotic polypharmacy). SGAPs are increasingly prescribed, combined in APP, in off‐label indications and with doses higher than the maximum recommended (Farhadian et al. 2011). In Rezaie, Nazari, Safari‐Faramani, et al. (2022), 73% of psychiatrists (*n* = 206) preferred other strategies before resorting to CLZ, even after two or three failed AP trials, and 55% (*n* = 155) admitted to delay CLZ even when other suitable alternatives were lacking. Clinicians often overestimate the effectiveness of these strategies or underestimate the effectiveness of CLZ (Agid et al. 2024) and inpatient psychiatrists tend to disregard both the guidelines and the suggestions of the outpatient teams (Oloyede et al. 2023).

Concerning the choice of multiple antipsychotic trials, in Jakobsen et al. (2022), an average of 4 trials occurred before the introduction of CLZ. Only 15.3% of patients (*n* = 20) were CLZ‐naïve and initiated CLZ over the 5‐year period of the study. In addition, the group of patients treated with CLZ consisted of significantly older patients and were considered ‘high‐needs’. In Daod et al. (2019), 53.3% of psychiatrists (*n* = 157) claimed to start CLZ according to the guidelines, but 33% (*n* = 97) only prescribed it after three or more failed AP trials. In Okhuijsen‐Pfeifer et al. (2019), although all clinicians considered CLZ as a third‐line medication, 47% of psychiatrists (*n* = 53) and 71% of nurse practitioners (*n* = 29) had patients who had already used more than three APs. In Gee et al. (2013) and Nielsen et al. (2009), 78% (*n* = 105) and 44.9% of clinicians (*n* = 44) would start CLZ after two unsuccessful AP trials, 14% (*n* = 19) and 30.6% (*n* = 30) after three, and 3% (*n* = 4) and 18.4% (*n* = 19) only after four, respectively. Additionally, in Gee et al. (2013), only 4% (*n* = 4) would start CLZ after one trial. In Tungaraza and Farooq (2015), 40.5% of clinicians (*n* = 98) preferred to try multiple antipsychotics and, in Farhadian et al. (2011), 80.0% (*n* = 94) would only consider CLZ after three unsuccessful trials. In Cirulli (2005), 44.8% (*n* = 26) prescribed CLZ after trialling two medications, 22.4% (*n* = 13) after more than two, 53.4% (*n* = 31) after another AP and only 15.5% (*n* = 9) would prescribe it as soon as possible.

Concerning long‐acting injectable antipsychotic (LAI AP) use, in Grant et al. (2024) and Cirulli (2005), 59% (*n* = 35) and 3.4% of psychiatrists (*n* = 2) would only prescribe CLZ after trying an LAI AP, respectively. In Taylor et al. (2000), 51.9% of patients (*n* = 296) had been treated with oral and LAI APs simultaneously before starting CLZ. Several cliniciansfavour this strategy because they consider it to be less burdensome for patients in terms of adverse effects and monitoring (Jakobsen et al. 2023).

Concerning APP, in Jakobsen et al. (2022), among 668 patients with SCZ, 49.4% (*n* = 330) were being treated with AP monotherapy and 43.0% (*n* = 285) with polypharmacy, of which 86.8% (*n* = 244) were with two APs and 13.2% (*n* = 38) were with more than three antipsychotics. In Grant et al. (2024), Tang et al. (2017) and Farhadian et al. (2011), 52% (*n* = 31), 16%–20% (*n* = 101–124) and 67.0% of clinicians (*n* = 79) preferred polypharmacy before introducing CLZ, respectively. In Nielsen et al. (2009), 75% (*n* = 75) favoured a FGAP before introducing CLZ, 64.7% (*n* = 64) APP with two APs and 15.2% (*n* = 15) augmentation with a mood stabiliser. In Ristic et al. 2021, CLZ was the only AP prescribed in 63.3% of cases (*n* = 102), but was often co‐prescribed with other antipsychotics and/or mood stabliser. In Grover, Balachander, et al. (2015), 61.3% of psychiatrists (*n* = 336) preferred CLZ after two trials in monotherapy, 17.3% (*n* = 95) preferred antipsychotics polypharmacy, 8.2% (*n* = 45) combined treatment with electroconvulsive therapy, and 10.0% (*n* = 55) antipsychotic polypharmacy with CLZ. In the latter case, the favoured drugs were: risperidone (33.6%, *n* = 184), amisulpride (32.3%, *n* = 177) and aripiprazole (12.0%, *n* = 66). However, in J. Kim et al. (2005), it was found that the use of anticholinergics and benzodiazepines was significantly higher with risperidone compared with CLZ. In Tang et al. (2017), prescribers with very ill patients (i.e., highest quartile of SCZ‐related hospitalisations) were less likely to resort to polypharmacy than prescribers in the lowest quartile (2% vs. 10%).

In Jakobsen et al. (2023), when clinicians were asked about the criteria for considering a patient a candidate for CLZ therapy, two aspects came up repeatedly: that CLZ is a third‐line therapy and that the patient had to be at least markedly ill (Clinical Global Impression Severity of Symptoms [CGISS] ≥ 5), low functioning (Global Assessment of Functioning [GAF] < 50) and in significant subjective distress. Still, the majority of psychiatrists admitted using CLZ far beyond that line and no significant differences were found between patients considered adequately or inadequately treated on either the GAF or the CGISS.

#### 3.1.9 Selection of Doses and Therapeutic Regimens

In Grover, Balachander, et al. (2015), the initial CLZ doses ranged from 12.5 mg/day (37.0% of psychiatrists, *n* = 203) to 25 mg/day (44.3%, *n* = 243) and the minimum maintenance dose was considered to be 100 mg/day or less (49.6%, *n* = 272) or 200 mg/day or more (15.7%, *n* = 86). The average dose used was 400 mg/day in Sloan et al. (1997) and 195.67 mg/day, with 53.8% of patients (*n* = 28) taking between 112.5 and 200 mg/day in Verma et al. (2020). In Davis et al. (2013), there were no significant differences between the average dose of CLZ used and all‐cause, non‐patient‐initiated, patient‐initiated, due to AEs and due to all reasons other than adverse effect discontinuations. The maximum dose was considered to be 400 mg/day [73% of psychiatrists (*n* = 400) in Grover, Balachander, et al. (2015)], 450 mg/day [47% (*n* = 16) in Cirulli (2005)], less than 600 mg/day [28.4% (*n* = 23) in Nielsen et al. (2009)], more than 600 mg/day [2.6% (*n* = 14) in Grover, Balachander, et al. (2015)] or up to 900 mg/day [21% (*n* = 17) in Nielsen et al. (2009) and 32% (*n* = 11) in Cirulli (2005)].

This diversity of practices and lack of consensus on starting and maintenance doses are cited as barriers to CLZ prescribing. In Moody and Eatmon (2019), one of the questions with the most incorrect answers by clinicians concerned the appropriate dose for starting CLZ therapy. In Ismail et al. (2018), uncertainty about regimens was cited as one of the main factors contributing to CLZ under‐utilisation. Thus, 13% of clinicians (*n* = 17) in Oloyede et al. (2023) consider the improvement of clinical pathways a priority.

In Lappin et al. (2022), the average duration of CLZ treatment in 14 patients was 48 months, with an average dose of 312.5 mg, and therapeutic drug monitoring was only recorded in one. In Nielsen et al. (2009), psychiatrists indicated that they would only wait an average of 4.9 months before considering CLZ ineffective and starting an augmentation strategy.

In Nielsen et al. (2009), when positive symptoms persisted, psychiatrists would: increase the dose of CLZ (39.8%, *n* = 35), add a SGAP (29.6%, *n* = 26), a FGAP (23.9%, *n* = 21) or a mood stabliser (4.6%, *n* = 4). In the case of persistent negative symptoms, psychiatrist would: add another AP (77.0%, *n* = 57), increase the dose of CLZ (9.5%, *n* = 7), add an antidepressant (AD) (8.1%, *n* = 6) or a MS (2.6%, *n* = 2). Aripiprazole (56.8%, *n* = 46), risperidone (16.1%, *n* = 13) and quetiapine (14.9%, *n* = 12) were the preferred antipsychotics for augmentation, zuclopenthixol (54.3%, *n* = 44), perphenazine (12.4%, *n* = 10) and haloperidol (12.4%, *n* = 10) were the preferred FGAPs and lamotrigine (56.3%, *n* = 27), valproate (23.3%, *n* = 16) and carbamazepine (6.3%, *n* = 3) were the preferred mood stabilsers.

#### 3.1.10 Influence of Current Clinical Status and CLZ as a Last Resort

In Jakobsen et al. (2023), according to psychiatrists, 40.7% of patients were not prescribed CLZ because of their current clinical status and, in Jakobsen et al. (2022), 1 of the 13 CLZ discontinuations was due to clinical stability. Some clinicians consider that once a certain stability has been achieved (e.g., absence of relevant suffering, maintenance of sleep and ability to derive some pleasure from life), no change in treatment is justified. Clinicians fear that changes in therapy could trigger clinical deterioration or the emergence of AEs without additional benefits. Some choose not to inform patients about CLZ, justifying themselves with the fact that they are primarily responsible for any negative consequences of treatment (Jakobsen et al. 2023).

In Jakobsen et al. (2023), in 70.4% of patients, psychiatrists considered that there are few possible initiatives to increase CLZ prescription, the majority believing that they were already well treated (59.1% of cases), treated as well as possible (18.2% of cases) or too compromised to be candidates for CLZ treatment (59.1% of cases).

CLZ is still seen as a medication of last resort, reserved for situations in which all other therapeutic alternatives have failed. This negative attitude is associated with a lower likelihood of prescription (Grover, Hazari, et al. 2015; Rezaie et al. 2022). In Rezaie, Nazari, Safari‐Faramani, et al. (2022), 35% of psychiatrists (*n* = 99) stated they were unable to properly assess CLZ's efficacy due to its various controversies. Such attitudes are often acquired and reinforced during medical training, through the transmission of subjective experiences from other professionals. This phenomenon varies greatly between institutions and is shaped by local values, culture and clinical practices (Rezaie et al. 2022). However, in Oloyede et al. (2023), only 3% of clinicians (*n* = 4) considered the promotion of a cultural change associated with CLZ (i.e., adoption of a positive attitude toward it and reduction of the stigma associated with it) as a priority.

By contrast, in Grant et al. (2024), 71% of clinicians (*n* = 51) advocated CLZ introduction in the first year after the diagnosis of the mental disorder and rejected the idea that it should be reserved as a last resort. In these contexts, there is usually support from the institutional leadership toward CLZ prescribing (Carroll et al. 2024).

### 3.2 Medication and Patient‐Related Barriers

In general, medication‐related barriers include adverse effects, tolerability and requirements for therapeutic monitoring—a concern of 14.7% of clinicians (*n* = 5) in Walker et al. (2024). Patient‐related barriers are associated with treatment burden, a dynamic and multidimensional concept that comprises the set of tasks required by therapy (regardless of the mental disorder itself) and that interfere with daily activities (e.g., time and travels required, physical burden resulting from AEs, psychosocial impacts) (Bogers et al. 2014; Tungaraza and Farooq 2015; Verdoux et al. 2025; Wilson et al. 2019).

According to Oloyede et al. (2023), medication and patient‐related barriers were the second most frequently mentioned theme by clinicians, accounting for 39% of responses (*n* = 33). Sub‐themes identified included adherence, blood monitoring, family and carer attitudes, patient attitudes, adverse effects and stigma. Below, we organise and explore barriers in more detail, using an adapted version of this subdivision.

#### 3.2.1 Adverse Effects

Concerns about potential CLZ‐induced adverse effects was identified as one of the most common barriers to its prescription by clinicians in Ismail et al. (2018) and Okhuijsen‐Pfeifer et al. (2019). Adverse effects are a cause of CLZ discontinuation or delay according to 40% of clinicians (*n* = 113) in Rezaie, Nazari, Safari‐Faramani, et al. (2022), 64.1% (*n* = 189) in Daod et al. (2019), 41% (*n* = 67) in Singh et al. (2019), 47% (*n* = 67) in Cotes et al. (2021), 78.3% (*n* = 76) in Moody and Eatmon (2019), 36% (*n* = 4) in Maryan et al. (2019) and 12% (*n* = 7) in Cirulli (2005). They also accounted for 55.6% cases (*n* = 10) of discontinuation in Lappin et al. (2022), 28% (*n* = 51) in Davis et al. (2013) and 9 out of 13 cases in Jakobsen et al. (2022). Psychiatrists' perceptions vary significantly. The belief that CLZ is dangerous, overestimation of its adverse effect prevalence and severity and unfamiliarity with their management often emerge as key factors for non‐prescription (Ismail et al. 2018; Mistry and Osborn 2011; Tungaraza and Farooq 2015). In Grover, Balachander, et al. (2015), 15.9% of clinicians (*n* = 87) considered CLZ tolerability to be superior to that of other antipsychotics, 45.3% (*n* = 248) considered it to be equivalent, and 38.9% (*n* = 213) considered it to be inferior. In Gee et al. (2013), 60% (*n* = 72) expressed concern about CLZ tolerability and, in Swinton and Ahmed (1999), 16% of TRS cases (*n* = 5) were not treated with CLZ because, according to psychiatrists, the risks outweighed the benefits. In Rezaie, Nazari, Safari‐Faramani, et al. (2022), 43.3% of clinicians (*n* = 122) admitted that they were not familiar with the safety profile of CLZ but, in Oloyede et al. (2023), only 7% (*n* = 8) emphasised the importance of investing in the development of facilitators to improve adverse effect management.

In Rezaie, Nazari, Safari‐Faramani, et al. (2022), 64% of psychiatrists (*n* = 180) regarded CLZ initiation as unsafe, although 72% (*n* = 203) stated that it did not increase the risk of death compared to other APs. In fact, in Davis et al. (2013), 13% of patients (*n* = 24) being treated with CLZ died. The most frequent causes were cardiovascular (58%, *n* = 13) and pulmonary (13%, *n* = 3), with an average age of 55.1 years at the time of death and an average duration of exposure to CLZ of 68.3 months. However, only 8% of deaths (*n* = 2) were directly attributed to CLZ: one by CLZ‐induced agranulocytosis (CIA) and the other by aspiration associated with CLZ‐induced constipation. On the other hand, there are contexts in which psychiatrists show less concern about adverse effects, largely due to their low frequency (Jakobsen et al. 2023). In Grant et al. (2024), more than 70% of clinicians disagreed that the risk of CIA (*n* = 52) or cardio‐metabolic effects (*n* = 47) should delay CLZ initiation. In Rezaie, Nazari, Safari‐Faramani, et al. (2022), less than 50% (*n* = 141) reported having experienced a serious AE in the previous 6 months.

In Wilson et al. (2019), pharmacists who had not received specific training on CLZ expressed concerns about its adverse effects. Yet, some emphasised that community dispensing of CLZ could actually increase patient safety because pharmacists knew their clients' clinical records, spent more time with them than hospital staff and had more opportunities to identify adverse effects. They also found it reassuring to be able to count on the support of the local mental health service to clarify any doubts.

In Maryan et al. (2019), 90% of psychiatrists (*n* = 10) considered collaboration with a pharmacist in the assessment of each patient to be a facilitator to the management. However, there were no significant differences between patients followed in pharmacist‐psychiatrist collaborative clinics and those followed in psychiatrists‐only clinics regarding clinical parameters such as glycated haemoglobin or haemoglobin A1C (HbA1C) values, body mass index (BMI), weight, total cholesterol (TC), low density lipoproteins (LDL), high density lipoproteins (HDL), triglycerides (TG), blood pressure, heart rate, and number of medications used to control adverse effects between baseline and end point. There was a slight reduction in HbA1c (−0.04 ± 0.25%) and TC levels (−15.73 ± 42.31 mg/dL) in collaborative clinics and TC values (−2.18 ± 13.97 mg/dL) in psychiatrist‐only clinics. The other indicators remained stable over the same period.

Moreover, patients are often reluctant or refuse CLZ initiation due to concerns about adverse effects according to 83.4% of psychiatrists (*n* = 246) in Daod et al. (2019), 62.8% (*n* = 344) in Grover, Balachander, et al. (2015), 71% (*n* = 91) in Gee et al. (2013) and 3 out of 9 cases of refusal by patients in Jakobsen et al. (2022). In Gee et al. (2013), patients showed concern about CLZ tolerability, in Taylor et al. (2000), 6.5% (*n* = 37) considered that its benefits not outweighed its disadvantages and, in Grover, Balachander, et al. (2015), 19.3% of psychiatrists (*n* = 106) claimed their patients viewed CLZ as a dangerous medication. On the contrary, in Verma et al. (2020) and Taylor et al. (2000), 50% (*n* = 26) and 87.0% of patients (*n* = 496), respectively, recognised that the benefits of CLZ clearly outweigh its undesirable effects. In Takeuchi et al. (2016), 68.0% (*n* = 68) believed CLZ to be a safe medication and 61.0% (*n* = 61) recognised their need for the treatment.

In Waserman and Criollo (2000), patients consistently perceived a worsening of nocturnal hypersalivation after trialling other antipsychotics and starting CLZ. Other than that, the majority of them did not notice any significant differences with CLZ. In Singh et al. (2019), only 4% of patients (*n* = 3) had experienced significant adverse effects. However, in Tungaraza and Farooq (2015), 45.5% of psychiatrists (*n* = 105) stated that their patients experienced adverse effects and, in Sharma et al. (2021), 75% of the patients who discontinued therapy (*n* = 12) pointed this as the main reason, 62.5% (*n* = 10) of whom had multiple adverse effects.

In some studies, patients reveal that they are unaware of adverse effects unless they have already experienced them (Blagden et al. 2020). In Angermeyer et al. (2001), when patients were asked about the long‐term risks of the therapy, a third admitted they did not know how to answer, a tenth had a vague idea about its potential impact on the haematopoietic system and a tenth said they feared dependence on CLZ or damage to organs. However, in Verma et al. (2020), patients were aware of effects such as haemorrhagic dyscrasias (97.4%, *n* = 47), CLZ‐induced weight gain (86.5%, *n* = 45), changes in lipid profile (80.8%, *n* = 42), CLZ‐induced hypersalivation (CIH) or sialorrhoea (90.4%, *n* = 47), hypotension (80.8%, *n* = 42), CLZ‐related hyperglycaemia or type 2 diabetes mellitus (75.0%, *n* = 39), CLZ‐induced seizures (63.5%, *n* = 33) and CLZ‐induced myocarditis (38.5%, *n* = 20). Carers showed greater knowledge of all adverse effects analysed, at least 90.4% of them (*n* = 47) knowing about each description, with the exception of myocarditis, whose risk was recognised by only 57.7% (*n* = 30).

In Qurashi et al. (2015), among the different AEs, patients rated as having the greatest negative impact on a 5‐point scale (5 being ‘very satisfied’), nocturnal hypersalivation [1.72 according to 84% (*n* = 47)], weight gaib [1.81 according to 57% (*n* = 32)], daytime hypersalivation [1.83 according to 41% (*n* = 23)] and constipation [1.86 according to 39% (*n* = 22)]. Increased appetite did not appear to be a cause for concern [2.87 according to 41% (*n* = 23)]. In Carroll et al. (2024), it was often pointed out that the presence of more or fewer symptoms turns out to be less important for the patients and their carers than their level of functionality and the number of hospitalisations.

In Sharma et al. (2021), patients who had cumulatively orthostatic hypotension, sedation and/or limb myoclonus rated CLZ as a less safe medication compared to other patients in relation to their antipsychotics. In Takeuchi et al. (2016), the aspects of CLZ treatment that caused patients the most anxiety were hypersalivation (63.0%, *n* = 63), sedation (51.0%, *n* = 51) and rebound psychosis (47.0%, *n* = 47). The aspects clinicians with experience in prescribing CLZ believed caused their patients the most anxiety were hypersalivation (66.7%, *n* = 38), sedation (49.1%, *n* = 28) and haematological monitoring (49.1%, *n* = 28). Clinicians with no experience in prescribing CLZ pointed to agranulocytosis (48.9%, *n* = 23), weight gain (38.3%, *n* = 18) and hypersalivation (34.0%, *n* = 16) as the most worrisome for patients. In Nielsen et al. (2009), clinicians admitted they considered agranulocytosis, cardiac complications and rebound psychosis to be much more problematic than their patients.

#### 3.2.2 Adherence to Haematological Monitoring

Non‐adherence to routine haematological monitoring or its expectation by clinicians is a barrier to CLZ treatment according to 74% of clinicians (*n* = 106) in Cotes et al. (2021), 74.2% (*n* = 72) in Moody and Eatmon (2019), 94% (*n* = 277) in Daod et al. (2019), 54.5% (*n* = 6) in Maryan et al. (2019), 62.5% (*n* = 65) in Takeuchi et al. (2016), 74% (*n* = 180) in Tungaraza and Farooq (2015), 74.8% (*n* = 410) in Grover, Balachander, et al. (2015), 85.0% (*n* = 100) in Farhadian et al. (2011) and 17.2% (*n* = 10) in Cirulli (2005). It also affected 69.2% and 41% of cases (*n* = 13) according to psychiatrists in Jakobsen et al. (2023) and Swinton and Ahmed (1999), respectively, and was the reason for CLZ therapy ineligibility in 6 out of 7 cases in Jakobsen et al. (2022). It was considered one of the three most common barriers in Okhuijsen‐Pfeifer et al. (2019) and in 30.6% of cases by non‐prescribing clinicians in Jakobsen et al. (2023).

In Taylor et al. (2000), 64% of patients (*n* = 365) accepted haematological monitoring as a necessary part of treatment and 4.7% (*n* = 27) even said they looked forward to it. However, in Verma et al. (2020), 46.2% of patients (*n* = 24) considered it distressing. Reluctance to perform blood sampling to stablish the patients' baseline values before CLZ initiation is one of the three most common barriers according to nurse practitioners in Okhuijsen‐Pfeifer et al. (2019) and occurs according to 60% (*n* = 45) and 85% of clinicians (*n* = 109) in Grant et al. (2024) and Gee et al. (2013), respectively. On the other hand, reluctance to routine monitoring throughout treatment occurs according to 84% (*n* = 63) and 92% of clinicians (*n* = 117) in Grant et al. (2024) and Gee et al. (2013), respectively, and 28.2% (*n* = 161) and 75% of patients (*n* = 18) in Taylor et al. (2000) and Swinton and Ahmed (1999), respectively.

Refusal is often associated with fear and pain during blood sampling and the exhaustiveness of the monitoring regimen (Carroll et al. 2024; Jakobsen et al. 2022). There is also variable understanding among patients as to the meaning of monitoring. In Taylor et al. (2000), 80.5% of patients (*n* = 485) claimed to have received sufficient information from clinicians or family members: 58.6% (*n* = 269) mentioned checking white blood cells, 10.5% (*n* = 48) mentioned protection against infections and 2.4% (*n* = 11) mentioned the risk of neutropenia. However, 12.4% (*n* = 57) gave an incorrect reason, and 17.4% (*n* = 99) said they did not know the reason for monitoring. In Blagden et al. (2020), despite often not understanding the reasons for monitoring, patients perceived CLZ favourably.

In Swinton and Ahmed (1999), acceptance of haematological monitoring appeared to be significantly associated with the patients' level of insight. Those who agreed to have the samples taken (*n* = 14) had an average Schedule for the Assessment of Insight score of 9.5, while those who refused it (*n* = 43) had an average score of 5.5.

In Cirulli (2005), 76% of clinicians (*n* = 26) who prescribed CLZ considered the monitoring to be useful in managing treatment. Some professionals recognise some positive aspects of monitoring regimens and even consider essential to see patients with some regularity: promote patient' holistic recovery, reinforcing their knowledge of CLZ and fostering a sense of responsibility and autonomy in relation to their health (Carroll et al. 2024; Blagden et al. 2020). This would help maintain engagement with services, improve therapeutic alliance, carry out regular physical health assessments and early identification of serious AEs (Cheng et al. 2024).

Yet, in Oloyede et al. (2023), only 9% (*n* = 11) thought it was essential to develop facilitators to improve adherence to monitoring. In Gee et al. (2013), 33% (*n* = 39) considered shortage of qualified staff as an obstacle to CLZ initiation and 82% (*n* = 97) proposed dedicated staff for obtaining baseline values as a facilitator. However, shortage of qualified professionals was not considered a relevant barrier by 72% of clinicians (*n* = 52) in Grant et al. (2024). Simplification of the haematological monitoring regimen (Jakobsen et al. 2023; Okhuijsen‐Pfeifer et al. 2019), including reduced monitoring frequency—according to 29.3% of clinicians (*n* = 58) in Grau‐López et al. (2020)—and eliminating the need to travel to laboratories—according to 70.0% (*n* = 68) in Moody and Eatmon (2019)—could be other key facilitators.

Hence, portable point‐of‐care (POC) devices for capillary haematological monitoring may be particularly valuable. In Bogers et al. (2014), inpatients and outpatients gave an average score of 4.8 and 3.0, respectively, when asked about the influence of the sampling method on the motivation to start CLZ (10 being ‘maximum influence’). Inpatients and outpatients also gave an average score of 8.7 and 6.9, respectively (0 being ‘preference for venous sampling’ and 10, ‘capillary sampling’). Capillary sampling was significantly preferred in relation to total burden, perceived pain and fear, convenience regarding timing, setting, and immediacy of results, anticipatory stress, dissatisfaction and in relation to delusional ideas (e.g., feeling that ‘blood was being sucked out of them’ or fears that the blood could be used for other purposes). Patients suggested that capillary sampling would have facilitated cooperation during acute psychotic phases. Clinicians also gave an average score of 8.9, and reasons included: greater acceptance from patients, increased motivation and cooperation, less activation of delusional ideas, greater scheduling flexibility and time savings.

Although psychiatrists recognise that POC devices can be a facilitator, some believe that this measure alone will not significantly increase prescription (Jakobsen et al. 2023). In Jakobsen et al. (2023), in 37.1% of cases, non‐prescribing clinicians highlighted the importance of initiatives focused on haematological monitoring, being more optimistic in this respect than prescribing clinicians.

#### 3.2.3 Patient Attitudes and Overall Adherence

Clinicians' concerns about adherence to treatment is one of the main reasons for not prescribing CLZ (Farhadian et al. 2011; Ismail et al. 2018). This barrier was mentioned by 19% of clinicians (*n* = 27) in Cotes et al. (2021), 82% (*n* = 242) in Daod et al. (2019), 11.3% (*n* = 11) in Moody and Eatmon (2019), 67.3% (*n* = 369) in Grover, Balachander, et al. (2015), 54% (*n* = 131) in Tungaraza and Farooq (2015), 53% (*n* = 17) in Swinton and Ahmed (1999) and in 22.2% of cases Jakobsen et al. (2023). Lack of adherence comprised 38.9% of CLZ discontinuations (*n* = 7) in Lappin et al. (2022), in 30.8% (*n* = 4) in Jakobsen et al. (2022) and 35% (*n* = 64) in Davis et al. (2013). However, Singh et al. (2019) showed a discontinuation rate of only 5% (*n* = 4). In Grant et al. (2024), 51% of clinicians (*n* = 39) mentioned that the need for readmission for CLZ initiation contributes somewhat frequently to CLZ delays, but 46% (*n* = 35) did not consider it an impeding factor. In Filia et al. (2012), poor adherence was perceived as a barrier to transition to GP shared‐care or private psychiatric settings, with a score of 2.1–2.7 (3 being ‘very important’).

Some psychiatrists consider that non‐prescribing clinicians (e.g., nurses) have more frequent and informal contacts with patients, which allows them to better capture patients' suffering and satisfaction. Prescribing clinicians tend to follow‐up a larger population, with shorter and more spaced‐out consultations, which can lead them to have a more positive or negative view than reality (Jakobsen et al. 2023).

#### 3.2.4 Patient Capacity for Cooperation

Cognitive impairment, difficulty understanding complex subjects or lack of insight are frequently identified barriers (Agid et al. 2024; Jakobsen et al. 2023). Some patients refuse CLZ because they feel that the responsibility associated with it is too demanding—as reported in 1 in 9 cases of refusal in Jakobsen et al. (2022)—or because they distrust the system and their carers (Agid et al. 2024). This criterion is often taken into account by psychiatrists, especially in the case of patients with markedly chaotic lifestyles or substance use disorders (Agid et al. 2024; Lappin et al. 2022). In Filia et al. (2012), patients' organisational capacity and ability to independently attend appointments and blood draws were rated with a score of 2.0–2.3 and 2.4 to 2.9, respectively (3 being ‘very important’) as facilitators to the transition to GP shared‐care or private psychiatric settings.

According to Davis et al. (2013), USA armed forces veterans eligible for disability compensation receive a score from 0% to 100%, with the number of benefits being proportional to the degree of disability. The average disability of patients who discontinued treatment due to poor adherence was 47%, while the average of those who maintained treatment was 61%, suggesting that higher levels of disability are associated with a lower risk of adherence‐related discontinuation.

#### 3.2.5 Patient Satisfaction

Several studies indicate a favourable trend with patients being satisfied with CLZ treatment: 92% (*n* = 44) in Grant et al. (2024), 58% (*n* = 58) in Takeuchi et al. (2016) and according to 65.4% of psychiatrists (*n* = 192) in Daod et al. (2019). Patients are either equally satisfied—20.5% (*n* = 18) in Sharma et al. (2021), 44.2% (*n* = 23) in Verma et al. (2020), 26.3% (*n* = 144) in Grover, Balachander, et al. (2015), 9.8% (*n* = 56) in Taylor et al. (2000)—or more satisfied with CLZ therapy—85% (*n* = 50) in Grant et al. (2024), 72% (*n* = 62) in Sharma et al. (2021), 53.8% (*n* = 28) in Verma et al. (2020), 67.3% (*n* = 369) in Grover, Balachander, et al. (2015), 89% (*n* = 50) in Qurashi et al. (2015), 30% (*n* = 24) in Angermeyer et al. (2001), 80% (*n* = 104) in Waserman and Criollo (2000), 86.1% (*n* = 491) in Taylor et al. (2000) and according to 71% of clinicians (*n* = 96) in Gee et al. (2013). Finally, 86% (*n* = 48) and 86% of patients (*n* = 112) considered CLZ to be better than previously used APs in Qurashi et al. (2015) and Waserman and Criollo (2000), respectively, and 88.6% (*n* = 505) and 73% (*n* = 95) intended to continue CLZ in Taylor et al. (2000) and Waserman and Criollo (2000), respectively.

In Taylor et al. (2000), when patients taking CLZ were asked about the positive aspects of previous treatment with FGAPs, 40% (*n* = 186) said they did not identify any benefit, and only 8.1% (*n* = 38) mentioned positive aspects (e.g., control of their disorder). Regarding negative aspects, 21.9% (*n* = 102) pointed to the ineffectiveness of the medications and 15.1% (*n* = 70) to their adverse effects. In Angermeyer et al. (2001), patients emphasised the fact that CLZ, compared to FGAPs, did not cause parkinsonism (27.5%, *n* = 22), akathisia (12.5%, *n* = 10), acute dystonia (12.5%, *n* = 10) and restlessness (11.2%, *n* = 9), as well as causing less sedation (8.3%, *n* = 7) and cognitive deficits (3.7%, *n* = 3). Patients' preference for CLZ over FGAPs was maintained after 6 months.

In J. Kim et al. (2005), patients taking CLZ obtained a positive mean score on the Drug Attitude Inventory (DAI)‐10 scale, reflecting a positive attitude toward CLZ and, in Sloan et al. (1997), patients gave CLZ treatment an average score of 27.4 on the Client Satisfaction Questionnaire (CSQ)‐8 scale, indicating high levels of satisfaction. In Takeuchi et al. (2016), 66.0% of patients (*n* = 66) believed that CLZ was an effective medication.

Among the reasons patients gave for their satisfaction, the symptomatic improvement in various domains stands out: thought domain [46% (*n* = 26) in Qurashi et al. (2015) and 68% (*n* = 89) in Waserman and Criollo (2000)], including a decrease in paranoia and delusions [Sharma et al. 2021, 65.4% (*n* = 34) in Verma et al. (2020)] and greater cognitive capacity [65.4% (*n* = 34) in Verma et al. (2020) and 5.0% (*n* = 4) in Angermeyer et al. (2001)]; perception domain, including fewer hallucinations [Sharma et al. 2021, 75.0% (*n* = 39) in Verma et al. (2020) and 18.7% (*n* = 15) in Angermeyer et al. (2001)]; mood domain [43% (*n* = 24) in Qurashi et al. (2015) and 65% (*n* = 84) in Waserman and Criollo (2000)], including fewer anxiety symptoms [Sharma et al. 2021, 73.1% (*n* = 38) in Verma et al. (2020) and 7.5% (*n* = 6) in Angermeyer et al. (2001)], depressive symptoms and suicidal ideation [71.2% (*n* = 37) in Verma et al. (2020) and 11.2% (*n* = 9) in Angermeyer et al. (2001)]; behavioural domain including improved behaviour toward others [63.5% (*n* = 33) in Verma et al. (2020)], ability to cope with stress [69.2% (*n* = 36) in Verma et al. (2020)] and to remain calmer [68.6% (*n* = 35) in Verma et al. (2020) and 27.5% (*n* = 22) in Angermeyer et al. (2001)]; sleep domain [Sharma et al. 2021, 80.8% (*n* = 42) in Verma et al. (2020), 48% (*n* = 27) in Qurashi et al. (2015), 27.5% (*n* = 22) in Angermeyer et al. (2001) and 72% (*n* = 93) in Waserman and Criollo (2000)]; and function [57.7% (*n* = 30) in Verma et al. (2020), 7.5% (*n* = 6) in Angermeyer et al. (2001) and 57% (*n* = 74) in Waserman and Criollo (2000)] and social skills [52% (*n* = 29) in Qurashi et al. (2015), 59% (*n* = 77) in Waserman and Criollo (2000) and 57% (*n* = 325) in Taylor et al. (2000)] domains.

Patients also highlighted the following as reasons for their satisfaction: clinical stability [71.2% (*n* = 37) in Verma et al. (2020), 5.0% (*n* = 4) in Angermeyer et al. (2001) and according to 82% of residents who prescribed CLZ (*n* = 58) in Singh et al. (2019)]; greater adherence to medication [73.1% (*n* = 38) in Verma et al. (2020) and 96% (*n* = 54) in Qurashi et al. (2015)]; reduction in the number of medications [65.4% (*n* = 34) in Verma et al. (2020)], better tolerability of CLZ [4% (*n* = 48) in Taylor et al. (2000)]; improvement in the way others relate to them [73.1% (*n* = 38) in Verma et al. (2020)]; capacity to be discharged from hospital, live in a hostel and get a job [52.9% (*n* = 302), 42.9% (*n* = 245) and 7.0% (*n* = 40) in Taylor et al. (2000), respectively]; and the subjective perception of feeling the same [30.8% (*n* = 16) in Verma et al. (2020) and 5.0% (*n* = 4) in Angermeyer et al. (2001)] or better than before developing the disorder [48.1% (*n* = 25) in Verma et al. (2020) and 35.4% (*n* = 202) in Taylor et al. (2000)]; and improved quality of life [68% (*n* = 38) in Qurashi et al. (2015) and 75% (*n* = 97) in Waserman and Criollo (2000)].

In Qurashi et al. (2015), improved quality of life had the greatest hedonic impact, with a score of 4.37 on a 5‐point scale (5 being ‘very satisfied’). Although improved social skills were the second most frequently reported improvement compared to previous antipsychotics, its hedonic impact was lower than increased activity levels and improved attention and alertness (4.24 vs. 4.35 vs. 4.31, respectively).

In Angermeyer et al. (2001), 43.7% of patients (*n* = 35) expected their mental state to deteriorate if they stopped taking CLZ and 20.0% (*n* = 16) expressed a fear of relapse. Only 7.5% (*n* = 6) believed that their condition would improve with CLZ discontinuation. Patients in Verma et al. (2020) agreed that their clinical condition would worsen if they discontinued CLZ (26.9%, *n* = 14) and that they felt somewhat dependent on this medication (38.5%, *n* = 20), but did not believe there was a need to change or discontinue therapy, given the benefits (44.2%, *n* = 23) and their overall distress (46.2%, *n* = 24). Also, 48.1% of patients (*n* = 25) felt that CLZ had been started too late.

Patients who admit being less satisfied with CLZ tend to be a minority: 32.7% (*n* = 17) in Verma et al. (2020), 1.5% (*n* = 1) in Sharma et al. (2021), 6.4% (*n* = 35) in Grover, Balachander, et al. (2015), 2.7% (*n* = 15) in Taylor et al. (2000), 19% (*n* = 25) in Gee et al. (2013), 66.0% (*n* = 66) in Nielsen et al. (2009) and according to 25.8% of clinicians (*n* = 76) in Daod et al. (2019). In Sharma et al. (2021), satisfaction, improved quality of life and changes in social skills did not differ significantly between patients using CLZ and those taking other APs and, in Taylor et al. (2000), 11.1% of patients (*n* = 63) reported that their lives had not changed with CLZ. However, according to Cotes et al. (2021), no active CLZ prescriber (i.e., a clinician who had prescribed CLZ in the previous month) reported that their patients would be less satisfied with CLZ compared to other antipsychotics.

Sharma et al. (2021) identified three clusters of patients: 18.8% of patients (*n* = 13), considered CLZ to be less safe, showed equal or lower satisfaction and reacted negatively to haematological monitoring; 24.6% (*n* = 17) had more hospitalisations, almost all had undergone trials with risperidone and quetiapine, and were more tolerant of haematological monitoring; and 56.6% (*n* = 39) preferred CLZ, perceived it to be safer and more effective, had undergone fewer AP trials and hospitalisations, were less likely to use tobacco or marijuana, and more likely to have a higher academic degree.

Some patients are resistant to CLZ initiation—according to 31.6% of psychiatrists (*n* = 173) in Grover, Balachander, et al. (2015) and 60% (*n* = 146) in Tungaraza and Farooq (2015). There are various reasons for this: they are not fully convinced of its efficacy [69% (*n* = 52) in Grant et al. (2024) and 62% (*n* = 80) in Gee et al. (2013)] and its advantages over other medications [70% (*n* = 53) in Grant et al. (2024); Rubio and Kane 2019], do not want to introduce something ‘foreign’ into their body (Carroll et al. 2024), reject taking medication regularly (Rezaie et al. 2022) or prefer to stick with known medications (Agid et al. 2024; Okhuijsen‐Pfeifer et al. 2019). Many refuse to replace an LAI AP with CLZ, even when they show only a partial response (Agid et al. 2024).

In Cirulli (2005), 97% of psychiatrists (*n* = 33) who prescribed CLZ discussed this treatment option with patients and their carer. However, some clinicians claim that explaining CLZ treatment is too time‐consuming (Agid et al. 2024) and that there is a shortage of clear and accessible educational materials (Moody and Eatmon 2019). Others report felling poorly trained in providing medical information before prescribing (Walker et al. 2024). According to Farhadian et al. 2011, and 20% of clinicians (*n* = 24) in Oloyede et al. (2023), it is important to develop facilitators to improve communication.

#### 3.2.6 Family and Carer‐Related Attitudes

The difficulty in convincing carers of the need to start CLZ contributes to its delay—according to 67% of clinicians (*n* = 48) in Grant et al. (2024), 50% (*n* = 141) in Rezaie, Nazari, Safari‐Faramani, et al. (2022) and 56.2% (*n* = 127) in Tungaraza and Farooq (2015). Furthermore, the lack of carer satisfaction, knowledge and collaboration regarding CLZ titration, haematological monitoring and surveillance of signs and symptoms of potential AEs represents an additional challenge for psychiatrists (Rezaie et al. 2022).

In Verma et al. (2020), carers reported that patients were dependent on CLZ (32.7%, *n* = 17), more sedated (57.7%, *n* = 30), lethargic, sad or weak (34.6%, *n* = 18) and with cognitive effects such as forgetfulness (34.6%, *n* = 18), distraction (21.2%, *n* = 11) or inability to maintain concentration (26.9%, *n* = 14). However, they believed that the benefits of CLZ outweighed its adverse effects (44.2%, *n* = 23), that patients were better able to perform everyday tasks (78.3%, *n* = 38), that taking CLZ was equal to (53.8%, *n* = 28) or better (48.1%, *n* = 25) than taking other APs, that there was no need to change the therapy in view of its beneficial effects (53.8%, *n* = 28) and in view of the disorder as a whole (67.3%, *n* = 35), and that CLZ had been started too late (71.2%, *n* = 37). Also, in terms of caregiver outcomes, carers reported a reduction in caregiver needs (65.4%, *n* = 34), personal caregiver distress (71.2%, *n* = 37) and tension (78.8%, *n* = 41), caregiver burden (73.1%, *n* = 38), time spent in caregiving (75.0%, *n* = 39) and treatment cost (51.9%, *n* = 27).

In Angermeyer et al. (2001), no statistically significant differences were found between the evaluations of patients and their carers regarding the positive effects of CLZ. Carers highlighted the overall improvement in the patients' condition and its calming effect. However, the majority feared a clinical deterioration if treatment was interrupted—a fear that contrasted with the perception of patients, only a third of whom shared this concern. The adverse effects that carers were most concerned about were those that were visible (e.g., weight gain), while patients were more concerned about symptoms that were less visible but strongly affected their well‐being and functioning (e.g., CIH and CIC). Around a third of carers showed fear of internal organ damage caused by long‐term CLZ treatment. Compared to FGAPs, carers highlighted the absence of extrapyrimidal side effects and the lower frequency of akathisia, sedation and cognitive deficits.

### 3.3 Administrative‐Related Barriers

Administrative and institutional barriers are related to lack of institutional experience, support and tools, and include: limited human and financial resources, shortage of facilities (e.g., inpatient beds for CLZ initiation, outpatient haematological monitoring), fragmentation of services (i.e., inpatient, outpatient and pharmacies), lack of community support (e.g., transport for patient appointments and lab visits, lack of systems to ensure adherence), appointments that are too brief and constant turnover of prescribers, shortage of CLZ itself at hospital level and administrative difficulties in registering patients on computer systems (Agid et al. 2024; Carroll et al. 2024; Oloyede et al. 2023; Rezaie et al. 2022; Rezaie et al. 2022b; Rubio and Kane 2019; Sharma et al. 2021; Takeuchi et al. 2016).

In Davis et al. (2013), administrative barriers contributed to CLZ discontinuation in 19% of cases (*n* = 35). In Oloyede et al. (2023), these barriers were the most frequently mentioned by clinicians (41%, *n* = 35), including sub‐themes such as blood monitoring, CLZ initiation, patient information, resources and treatment pathways. Below, we explore barriers in more detail, using an adapted version of this subdivision.

#### 3.3.1 Clozapine Initiation and Clozapine‐Specialised Clinics

In Tungaraza and Farooq (2015), 42.9% of psychiatrists (*n* = 104) considered CLZ initiation to be a cumbersome process. Initiation of CLZ therapy is an administrative barrier when there are no community teams capable of carrying out titration (i.e., forcing patients to start CLZ in an inpatient setting) (Oloyede et al. 2023) and/or when there are no inpatient beds available for this purpose. In this study, 25.1% of psychiatrists (*n* = 57) considered initiating CLZ in the community to be unsafe, and 12.3% (*n* = 28) did not know if this practice was safe. However, the requirement for inpatient initiation, whenever mandatory, was pointed out as a factor delaying CLZ therapy by clinicians in Ismail et al. (2018), 50% of clinicians (*n* = 36) in Grant et al. (2024), 32% (*n* = 78) in Tungaraza and Farooq (2015) and 33% (*n* = 38) in Gee et al. (2013).

Inpatient places are often limited by shortage of beds. Some emphasise the need to increase this capacity (Ismail et al. 2018; Walker et al. 2024), which was considered a facilitator by 25% (*n* = 28) and 69% of clinicians (*n* = 80) in Okhuijsen‐Pfeifer et al. (2019) and Gee et al. (2013), respectively. Nevertheless, the majority of CLZ candidates are not in an acute decompensation phase, not being prioritised for access (Tungaraza and Farooq 2015) and the need for readmission can lead to patient refusal according to 54% of participants (*n* = 68) in Gee et al. (2013). The latter accounted for 1 of 9 refusals in Jakobsen et al. (2022).

There are also advocates of CLZ initiation in a community setting. In Grant et al. (2024), 93% of clinicians (*n* = 62) considered CLZ initiation in the community to be safe and, as such, the existence of professionals dedicated to CLZ initiation in these settings was seen as a facilitator by 81% (*n* = 51). In Nielsen et al. (2009), 76.0% (*n* = 75) stated that they would initiate CLZ in the community, although only 50.0% (*n* = 49) had already done so. In Verma et al. (2020), 84.6% of patients (*n* = 44) started CLZ on an outpatient basis.

In some regions, there are regional tertiary clinics with specialised CLZ services for this purpose. Patients attend on an appointment basis and, at each visit, are clinically assessed and monitored by multidisciplinary teams. Information is then provided to the patient's attending psychiatrist, ensuring continuity of care and facilitating the initiation and follow‐up of therapy (Sloan et al. 1997). Their availability was considered a facilitator by psychiatrists in Ismail et al. (2018), 60.4% of clinicians (*n* = 178) in Daod et al. (2019), 70.0% (*n* = 68) in Moody and Eatmon (2019), and 55% of psychiatrists (*n* = 62) and 44% of nurses (*n* = 18) in Okhuijsen‐Pfeifer et al. (2019). In Gee et al. (2013), the existence of a day hospital dedicated to CLZ initiation and of specific staff for its initiation and monitoring in outpatient settings was considered to be useful by 80% (*n* = 94) and 82% of clinicians (*n* = 96), respectively. In Grant et al. (2024), 78% (*n* = 56) did not consider the absence of such services to be a direct cause of CLZ delay, although more than 60% (*n* = 40) considered it would facilitate the process.

In Grover, Balachander, et al. (2015) and Tungaraza and Farooq (2015), 6.8% (*n* = 37) and 56.5% of clinicians (*n* = 130), respectively, said access to CLZ clinics varies depending on the region. Logistical issues were also mentioned by 70% (*n* = 68) in Moody and Eatmon 2019. However, in Tungaraza and Farooq (2015), no statistically significant correlation was found between the number of CLZ prescriptions in the previous 6 and 12 months and clinicians' access to such clinics. Similarly, in Daod et al. (2019), although 19.5% of psychiatrists (*n* = 47) had access to dedicated CLZ services, only 36.9% of them (*n* = 17) said they initiated therapy according to the guidelines, compared to 58.9% (*n* = 146) without access to such services. Psychiatrists with access tended to prescribe other APs more often (53.2%, *n* = 25 vs. 28.6%, *n* = 71), although they did not resort to APP as frequently as the second group (8.5%, *n* = 4 vs. 24.1%, *n* = 60). In Moody and Eatmon (2019), there were also no significant differences in prescription rates between institutions with and without CLZ clinics, or between clinics that had a pharmacist and those that did not.

#### 3.3.2 Lack of Resources

In Tungaraza and Farooq (2015) and Swinton and Ahmed (1999), 24.2% (*n* = 55) and 16% of clinicians (*n* = 5), respectively, felt they did not have the necessary resources to prescribe CLZ. In Oloyede et al. (2023), 26% (*n* = 31) stated it was essential to identify facilitators to overcome this barrier. In Moody and Eatmon (2019), 3.1% (*n* = 3) mentioned lack of access to health services as a barrier, which also accounted for 1 out of 7 cases considered ineligible for CLZ therapy in Jakobsen et al. (2022). In Rezaie, Nazari, Safari‐Faramani, et al. (2022), around 50% of psychiatrists (*n* = 141) said that they did not have adequate facilities for monitoring and managing patients on CLZ. Although 65% (*n* = 183) had access to crisis intervention and home support teams, over 90% (*n* = 254) reported that there were no specialised clinics in their workplace.

This is more evident in rural or semi‐rural areas, where there are no dedicated CLZ services, when general practitioners (GPs) do not take responsibility for haematological monitoring or patient follow‐up, accessing laboratory and medical centres is difficult and patients have to travel long distances to hospitals and carry out monitoring outside of working hours which leads to financial costs, difficulties coordinating schedules and anxiety (Ismail et al. 2018; Oloyede et al. 2023; Rezaie et al. 2022). Access can also be conditioned by extraordinary circumstances, such as when patients go on holidays or as occurred during the COVID‐19 pandemic (Agid et al. 2024; Filia et al. 2012). Therefore, some psychiatrists feel isolated and express concern about fulfilling the blood sampling frequency required by guidelines and about treatment safety and feasibility, making them less inclined to prescribe (Jakobsen et al. 2023; Rezaie et al. 2022). In Farhadian et al. (2011), 70% of clinicians (*n* = 83) considered that greater local support (i.e., patient enrolment, monitoring and management of psychotic symptoms) would contribute to an increase in CLZ prescribing. Although specialised clinics and interdisciplinary programmes have proven useful, a lack of resources limits their development and implementation (D. L. Kelly et al. 2017).

In Rezaie, Nazari, Safari‐Faramani, et al. (2022), 63% of psychiatrists (*n* = 178) reported that there was a limit to the number of patients who could start CLZ treatment due to financial constraints. Barriers include the financial burden of purchasing the medication (i.e., in cases where it is not reimbursed by the health system or insurance) and the means of transport needed to access health services (Carroll et al. 2024; D. L. Kelly et al. 2017). In Filia et al. (2012) not having health insurance was also considered a barrier in the transition of patients to private psychiatric care with a score between 1.6 and 2.9 (3 being ‘very important’). Concerns go beyond the patient level, encompassing financial costs linked to service coordination, training professionals to perform blood draws and maintenance of physical spaces intended for this purpose (Carroll et al. 2024; D. L. Kelly et al. 2017). A limited number of prescribers was also pointed out by 7.2% (*n* = 7) in Moody and Eatmon (2019). In Grau‐López et al. (2020) and Moody and Eatmon (2019), 5.8% (*n* = 12) and 3.1% (*n* = 3), respectively, suggested allowing CLZ prescription by GPs or advanced practice registered nurses.

#### 3.3.3 The Roles of Different Healthcare Professionals

There is uncertainty about the capacity of health services, as they are currently organised, to ensure adequate monitoring of CLZ treatment (Walker et al. 2024). In Blagden et al. (2020), professionals reported that responsibilities and roles of the different teams involved in CLZ treatment were not well defined. Neither group considered itself responsible for cardio‐metabolic monitoring and neither could clearly identify who should take on this responsibility: the physical health team saw monitoring as the responsibility of the psychiatrists, given that CLZ is an antipsychotics and that adverse effects should be monitored by the prescriber, while the psychiatrists considered that, since the physical parameters were extensive, this task would fall to the physical health team. Both groups recognised the benefit CLZ clinics, integrating mental and physical health professionals.

In Filia et al. (2012), one of the barriers identified to the transition of patients to the private sector was the less likelihood of such services to assume accountability for poor adherence to treatment, obtaining a score of 2.3–2.7 on a scale of 0–3 (3 being ‘very important’). It also mentioned the lack of support from public services for access to patients' clinical records and the refusal to take them back at the event of clinical deterioration. Thus, the guarantee of continued support from the public system in these situations was considered by some to be a necessary criterion for this transition.

There is a shortage of centralised infrastructure to coordinate CLZ treatment (e.g., patient and caregiver education, access to laboratory tests, medical consultations and monitoring of therapeutic adherence) (D. L. Kelly et al. 2017). The creation of a specific care coordinator responsible for monitoring the therapeutic process was mentioned by 53% (*n* = 156) and 58.7% of clinicians (*n* = 57) in Grant et al. (2024) and Moody and Eatmon (2019), respectively. In the latter study, involvement of nurses or pharmacists in patient follow‐up was also suggested by 4.1% of clinicians (*n* = 4).

The dispensing of CLZ itself can be an obstacle. CLZ is considered to be a highly specialised medication, and traditionally treatment was started in a hospital inpatient setting, under the responsibility of a psychiatrist to allow for more rigorous medical monitoring (Filia et al. 2012). Patients had to see the hospital psychiatrist regularly to obtain a prescription, which could then only be filled at a hospital pharmacy (Wilson et al. 2019). There was also the need for haematological tests to be immediately available when CLZ was delivered, making access to treatment more difficult (Ismail et al. 2018).

However, in some countries, after discharge, CLZ treatment can continue to be managed in public community services, on a GP shared‐care basis (maintaining regular follow‐up by a psychiatrist), or by definitive discharge from public services for exclusive private follow‐up. In these contexts, GPs can only prescribe the maintenance dose set by the psychiatrists (Filia et al. 2012). In the UK and New Zealand, this happens after 18 weeks of initial treatment under psychiatric supervision and with clinical and haematological stability (Wilson et al. 2019).

In some settings, the patient has the opportunity to fill a CLZ prescription at a registered community pharmacy (Filia et al. 2012; Wilson et al. 2019). According to some professionals, it is advantageous for patients to be able to purchase the medication from community pharmacies during the titration phase (Moody and Eatmon 2019). In Filia et al. (2012), access to pharmacies able to dispense CLZ obtained an average score of 2.7–2.9 (3 being ‘very important’) as to the transition of patients from public community mental health services to GP shared‐care or private care settings.

In Wilson et al. (2019), of the pharmacies analysed that did not have a CLZ dispensing service, 75.6% (*n* = 93) had never been approached about this possibility, and 50.4% (*n* = 62) had never considered it. The most frequently cited barrier was the lack of demand from consumers (52.5%, *n* = 64) as the low caseload could be troublesome to maintain pharmacists' professional competence. Other barriers included administrative delays in implementing the service, reluctance of local mental health services to refer eligible patients and doubts about where to look for information about implementation steps and the challenges of dealing with patients with SCZ (e.g., developing a therapeutic relationship, fears of potential aggressiveness) (Agid et al. 2024; Wilson et al. 2019).

In general, pharmacists thought that dispensing CLZ in community pharmacies would bring significant benefits for patients and that pharmacies would become more involved in mental health issues and expand their professional role. Facilitators would be more demand from users, encouragement of referrals from mental health services, dedicated administrative channels and protocols for managing risk situations (e.g., referral in cases of lack of adherence or haematological changes) (Wilson et al. 2019).

In Gee et al. (2013), pharmacists showed familiarity with the NICE guidelines for the treatment of SCZ (68%, *n* = 15 vs. 27% of doctors, *n* = 21 vs. 36% of other professionals, *n* = 14), were confident about CLZ efficacy (mean score of 8.2 vs. 7.9 for doctors vs. 7.4 for other professionals), reported a lower proportion of patients dissatisfied with the treatment (9%, *n* = 2 vs. 20% of doctors, *n* = 15 vs. 21% of other professionals, *n* = 8), and identified a greater number of eligible patients not being treated (58%, *n* = 7 vs. 47% of doctors, *n* = 25 vs. 38% of other professionals, *n* = 10). Pharmacists gave greater importance to the existence of administrative resources and additional clinical staff to facilitate prescribing (74%, *n* = 17 vs. 57% of doctors, *n* = 43 vs. 51% of other professionals, *n* = 21).

In Maryan et al. (2019), no significant differences were found between pharmacist‐psychiatrist collaborative clinics and psychiatrists‐only clinics regarding the number of APs, total number of psychotropic medications, doses of CLZ, emergency room visits and treatment discontinuation rates. Significantly more pharmacological (71 vs. 19) and non‐pharmacological (154 vs. 3) interventions were recorded in the collaborative clinics compared with the psychiatrists‐only clinics, respectively.

#### 3.3.4 Communication Among Healthcare Professionals

In Tungaraza and Farooq (2015), 39% of psychiatrists (*n* = 95) pointed fragmentation of services as a barrier to CLZ prescribing. Lack of adequate communication channels between clinicians and the various health services is a barrier to CLZ prescription (Ismail et al. 2018). This problem is particularly evident in prisons and forensic units. Many of these institutions operate independently and have scarce psychiatric services, which compromises continuity of care, often leading to the neglect of routine psychiatric follow‐up. There is also a lack of specific studies on CLZ use in these populations (D. L. Kelly et al. 2017; Rubio and Kane 2019).

Some strategies mentioned by clinicians included formal weekly meetings with the whole team (in order to develop collaborative work and align expectations and messages transmitted to the patient), occasional informal contact between team members whenever necessary (Carroll et al. 2024) and a pre‐defined plan to ensure that patients are followed‐up when their clinicians are absent (Moody and Eatmon 2019). Another obstacle mentioned to CLZ prescribing is the recent transfer of patients to the public sector after years of treatment in the private sector, often without adequate documentation on previous treatments (Lappin et al. 2022). In this regard, it has been suggested the creation of a simple platform to record the timelines of previously trialled treatments and their responses (Oloyede et al. 2023).

#### 3.3.5 Lack of Time and Legal Issues

Limited time available dedicated to CLZ initiation on an outpatient basis is also a frequently mentioned barrier and increasing such time was considered a facilitator by 51% of psychiatrists (*n* = 57) and 49% of nurse practitioners (*n* = 20) in Okhuijsen‐Pfeifer et al. (2019). Some clinicians report fears about possible legal repercussions and the impact these may have on their professional reputation, especially as some carers and patients tend to interpret the emergence of AEs as a sign of medical malpractice (Torrey and Lieberman 2024; Rezaie et al. 2022). This concern is particularly related to the perceived lack of legal support from institutions (Rezaie et al. 2022).

A summary of the analysis of the 53 eligible studies is compiled in Table 1.

**TABLE 1:** Summary of eligible studies on clozaphobia.

Authors (year)	Title	Country	Study design	Sample size	Sample characteristics
Walker et al. (2024)	Survey on barriers to psychiatrists' use of clozapine for young people in Scotland and suggestions for reducing these	Scotland, United Kingdom	Cross‐sectional survey	68 psychiatrists working In child and adolescent mental health services. *Response rate of* 33.8% (*n* = 34).	*Length of practice* Consultants: 67.6%; higher trainee: 23.5%; speciality doctor: 5.9%; retired: 2.9%. *Workplace* Inpatient: 14.7%; outpatient: 44.1%; combined: 32.3%; other: 8.8%.
Grant et al. (2024)	Psychiatrists' views on clozapine prescribing in Ireland	Ireland, European Union	Cross‐sectional survey	275 consultant psychiatrists practising in Ireland. *Response rate of* 28.0% (*n* = 77).	*Length of practice* 0–5 years: 10.4%; 6–10 years: 16.9%; 11–15 years: 18.2%; 16–20 years: 22.1%; > 20 years: 32.5%. *Workplace* Inpatient: 6.5%; outpatient: 15.6%; combined: 71.4%; general hospital: 6.5%.
Agid et al. (2024)	Overcoming the barriers to identifying and managing treatment‐resistant schizophrenia and to improve access to clozapine: A narrative review and recommendation for clinical practice	—	Narrative review	—	—
Carroll et al. (2024)	Multi‐level barriers and facilitators to implementing evidence‐based antipsychotics in the treatment of early‐phase schizophrenia	Michigan, Oregon, Oklahoma, Florida and Illinois, United States of America	Semi‐structured interviews	16 constituents associated with early psychosis intervention network (EPINET) clinics and the recovery from early psychosis program (REPP).	*Workplace* Prescribers: 12.5%; non‐prescribing clinicians: 31.3%; administrators: 18.8%; caregivers: 18.8%; clients: 18.8%.
Shangraw et al. (2024)	Medical, psychiatric, and sociodemographic predictors of clozapine initiation at an academic medical center	United States of America	Combined cohort and case‐control study	Cohort study: Hospitalised patients with schizophrenia or schizoaffective disorder, 59 prescribed clozapine and 477 not prescribed. Case‐control study: 40 hospitalised patients prescribed clozapine and 47 not prescribed.	—
Wu et al. (2024)	Safety of benign ethnic neutropenia guidelines in clozapine treatment: A canadian prospective	Canada	Retrospective chart review	41 patients from the center for addiction and mental health on clozapine treatment following BEN guidelines	—
Oloyede et al. (2023)	What are the barriers and facilitators of clozapine use in early psychosis? A survey of UK early intervention clinicians	England, United Kingdom	Cross‐sectional survey	Approximately 700 clinicians from 35 early intervention in psychosis (EIP) services. *Response rate of* 18% (*n* = 123).	*Length of practice* 0–5 years: 26.8%; 6–10 years: 17.9%; 11–20 years: 25.2%; > 20 years: 30.1%. *Workplace* Nurse: 51.2%; doctor: 19.5%; psychologist: 6.5%; social worker: 6.5%; other: 9.8%; consultant psychiatrist: 4.9%; pharmacist: 1.6%.
Jakobsen et al. (2023)	Non‐prescribing of clozapine for outpatients with schizophrenia in real‐world settings: The clinicians' perspectives	Denmark, European Union	1^st^phase: Cross‐sectional survey; 2^nd^phase: Semi‐ structured interviews	1^st^phase: Clinicians of 43 clozapine‐eligible, yet clozapine‐*naïve*, outpatients with schizophrenia. *Response rate of* 83% (*n* = 39 outpatients); 2^nd^phase: 10 of those psychiatrists.	*Workplace* Senior psychiatrists: 38.2%; nurses: 50.0%; other health‐related training: 11.8%.
Jakobsen et al. (2022)	Antipsychotic prescribing practices for outpatients with schizophrenia and reasons for non‐clozapine treatment—data from a Danish quality assessment audit	Denmark, European Union	Retrospective case note review	668 patients with schizophrenia and outpatient affiliation with the mental health services of region Zealand east	—
Rezaie et al. (2022)	Exploration of the barriers to clozapine prescribing in patients with treatment‐resistant schizophrenia: A qualitative study	Iran	Semi‐structured interviews	12 psychiatrists with at least 3 years of experience prescribing clozapine to patients with TRS.	*Length of practice* Less than 15 years: 52.2%; 15 years or more: 47.8%. *Workplace* With academic position: 17.5%; without academic position: 82.5%.
Lappin et al. (2022)	Underuse of recommended treatments among people living with treatment‐resistant psychosis	New South Wales, Australia	Retrospective case note review	36 patients with treatment resistant psychosis from the tertiary referral service for psychosis	—
Parkes et al. (2022)	Patients' experiences of clozapine for treatment‐resistant schizophrenia: A systematic review	London, England, United Kingdom	Systematic review	—	—
Rezaie, Nazari, Safari‐Faramani, et al. (2022)	Iranian psychiatrists' attitude toward clozapine use for patients with treatment‐resistant schizophrenia: A Nationwide survey	Iran	Cross‐sectional survey	306 psychiatrists registered with the Iranian psychiatrists association. *Response rate* of 92.2% (*n* = 282).	*Length of practice* 0–5 years: 16.7%; 6–10 years: 58.3%; 11–15 years: 16.7%; 16–20 years: 8.3%. *Workplace* Governmental practice: 41.7%; private practice: 16.6%; both: 41.7%.
Cotes et al. (2021)	Comparison of attitudes, comfort, and knowledge of clozapine among two diverse samples of United States of America psychiatrists	United States of America	Cross‐sectional survey	550 members of the American association of community psychiatrists; *response rate of* 10.3% (*n* = 57). 86 South‐eastern state Conference's attendees (SSCA). Total of 143 surveys.	*Length of practice* Trainee: 6.3%; post‐resident: 93.7%. *Workplace* Forensic: 2.1%; inpatient: 25.2%; outpatient: 74.1%.
Howes et al. (2021)	Treatment resistance in psychiatry: State of the art and new directions	—	Narrative review	—	—
Ristic et al. (2021)	Prescription attitudes and practices regarding clozapine among Serbian psychiatrists: Results of a nationwide survey	Serbia	Structured interviews	302 psychiatrists from three university clinics, three psychiatric hospitals and seven psychiatric departments of general hospital. *Response rate of* 53.3% (*n* = 161).	*Length of practice* 0–5 years: 19.8%; 6–10 years: 8.1%; 11–15 years: 18.0%; > 15 years: 51.5%; missing data: 2.4%. *Workplace* Outpatient: 46.0%; inpatient: 50.2%; missing data: 3.7%. University clinic: 36.6%; psychiatric hospital: 42.2%; general hospital: 21.1%.
Sharma et al. (2021)	Cluster analysis of clozapine consumer perspectives and comparison to consumers on other antipsychotics	Georgia, United States of America	Structured interviews	211 inpatients and outpatients from a public hospital in Atlanta, Georgia	*Workplace* Outpatient: 94.3%; inpatient: 5.7%.
Verma et al. (2020)	Attitude toward and experience with clozapine of patients and their caregivers after months of starting of clozapine	India	Cross‐sectional survey	52 patients with treatment resistance schizophrenia who had received clozapine for 3 months and 52 family caregivers of these patients.	—
Blagden et al. (2020)	A qualitative exploration of the barriers to and facilitators of clozapine monitoring in a secure psychiatric setting	England, United Kingdom	Semi‐structured interviews and focus groups	17 staff members and 6 patients from medium‐secure, low‐secure and step‐down wards from a secure mental health unit.	*Workplace* Doctors: 47.0%; junior doctors: 29.4%; general nurses: 11.8%; healthcare support workers: 11.8%.
Grau‐López et al. (2020)	Professional perception of clozapine use in patients with dual psychosis	Spain, European Union	Cross‐sectional survey	2000 attendees of the world association on dual disorders and the Spanish association of dual disorders congresses. *Response rate of* 10% (*n* = 199).	*Workplace* Physicians: 90.5%; psychologists: 9.5%. Mental health center: 38.4%; hospital: 39.9%; acute unit: 9.6%; addictions unit: 8.1%; day‐care center: 3.0%; residents: 1.0%.
Singh et al. (2019)	Comfort level and barriers to the appropriate use of clozapine: A preliminary survey of United States of America psychiatric residents	United States of America	Cross‐sectional survey	164 residents of psychiatry residency programmes affiliated with the accreditation council for graduate medical education.	*Length of practice* Junior residents (first 2 years of residency): 36.6%; senior residents (third year of residency or higher): 63.4%.
Rubio and Kane (2019)	How and when to use clozapine	—	Narrative review	—	—
Daod et al. (2019)	Psychiatrists' attitude toward the use of clozapine in the treatment of refractory schizophrenia: A nationwide survey	Israel	Cross‐sectional survey	620 registered psychiatrists in the country. *Response rate of* 47.6% (*n* = 295).	*Length of practice* Junior resident: 24.1%; senior resident: 15.3%; 0–5 years specialist: 43.1%; > 5 years specialist: 15.9%; other: 1.7%. *Workplace* Community clinic: 13.9%; outpatient clinic: 29.2%; private clinic: 2.0%; open ward: 20.0%; locked ward: 19.0%; combined ward: 13.9%.
Moody and Eatmon (2019)	Perceived barriers and facilitators of clozapine use: A national survey of veterans affairs prescribers	United States of America	Cross‐sectional survey	97 mental health providers within the veterans health administration who were clozapine prescribers.	*Length of practice* 0–5 years: 10.3%; 6–10 years: 18.5%; 11–15 years: 13.4%; > 15 years: 57.7%. *Workplace* Outpatient: 86.6%; inpatient: 13.4%. Doctor: 87.6%; advanced practice registered nurse: 9.3%; physician assistant: 3.1%.
Wilson et al. (2019)	Implementing a clozapine supply service in Australian community pharmacies: Barriers and facilitators	Australia	Stage one: cross‐sectional survey; stage two: semi‐structured interviews	Stage one: Approximately 5700 community pharmacies. *Response rate of* 4.6% (*n* = 265). Stage two: 129 community pharmacies from stage one which did not have a clozapine supply service. *Response rate of* 9.3% (*n* = 12).	—
Okhuijsen‐Pfeifer et al. (2019)	Differences between physicians' and nurse practitioners' viewpoints on reasons for clozapine underprescription	Flanders, Belgium and The Netherlands, European Union	Cross‐sectional survey	153 physicians and nurse practitioners through professional associations, academic hospitals, mental health centres and other platforms.	*Workplace* Psychiatrist: 57.5%; psychiatrist in training: 15.7; nurse practitioner: 26.1%; nurse practitioner in training: 0.7%.
D. L. Kelly and Love (2019)	Psychiatric pharmacist's role in overcoming barriers to clozapine use and improving management	—	Narrative review	—	—
Maryan et al. (2019)	Comparison of clozapine monitoring and adverse event management in a psychiatrist‐only and a clinical pharmacist‐psychiatrist collaborative clinic	United States of America	Stage one: prospective chart review; stage two: cross‐sectional survey	Stage one: patients prescribed clozapine in psychiatrist‐only and psychiatrist‐pharmacist collaborative clinic. Stage two: 16 psychiatrists, only 1 practising in a clozapine clinic. *Response rate of* 69.0% (*n* = 11).	*Workplace* Collaborative clinic: 50.0% patients; psychiatrist‐only clinic: 50.0% patients.
Ismail et al. (2018)	A qualitative exploration of clozapine prescribing and monitoring practices in the Arabian gulf countries	Qatar, Bahrain, Oman, Saudi Arabia, United Arab Emirates, Kuwait	Semi‐structured interviews	Psychiatrists, psychiatric residents, nurses, pharmacists and medical directors working in mental health hospitals, clinics and primary care settings.	*Length of practice* 0–9 years: 38.5%; ≥ 10 years: 61.5%. *Workplace* Psychiatrists: 53.8%; psychiatric residents: 7.7%; pharmacists: 30.8%; mental health nurses: 7.7%.
Kelly et al. (2017)	Addressing barriers to clozapine underutilisation: A National effort	United States of America			
Verdoux et al. (2018)	Prescriber and institutional barriers and facilitators of clozapine use: A systematic review	—	Systematic review	—	—
Tang et al. (2017)	Prescribing of clozapine and antipsychotic polypharmacy for schizophrenia in a large medicaid program	Pennsylvania, United States of America	Retrospective case note review	650 prescribers from the Pennsylvania's medicaid program prescribing at least one antipsychotic for 18–64 years and not dually eligible patients with schizophrenia	*Workplace* Psychiatrists: 83.5%; primary care provider: 5.7%; other: 10.8%
Sreeraj et al. (2017)	Clozaphobia: is avoidance of clozapine in diabetes warranted?	India	Case reports	9 patients with schizophrenia or schizoaffective disorder and comorbid diabetes mellitus treated with clozapine.	—
Takeuchi et al. (2016)	A questionnaire‐based study of the views of schizophrenia patients and psychiatric healthcare professionals in Japan about the side effects of clozapine	Japan	Cross‐sectional survey	106 patients at Okehazama, Kakamigahara and Numazu chuo hospitals on clozapine ≥ 1 month. *Response rate of* 94.3% (*n* = 100). 120 healthcare professionals. *Response rate of* 86.7% (*n* = 104).	*Workplace (patients)* Outpatient: 32.0%; inpatient: 68.0%. *Workplace (healthcare professionals)* Physicians: 51.9%; pharmacists: 48.1%
Grover, Balachander, et al. (2015)	Prescription practices and attitude of psychiatrists toward clozapine: A Survey of psychiatrists from India	India	Cross‐sectional survey	3381 Indian psychiatrists. *Response rate of* 16.2% (*n* = 548).	*Workplace* Faculty position in government institutes: 26.1%; private practice: 22.4%; faculty position and private practice: 20.8%; senior resident: 18.8%; faculty position in private institutes: 6.8%; trainee resident: 5.1%.
Grover, Hazari, et al. (2015)	Delay in initiation of clozapine: A Retrospective study from a tertiary care hospital in North India	India	Retrospective case note review	200 patients with schizophrenia, schizoaffective disorder or psychosis NOS in a governmental tertiary care center.	—
Tungaraza and Farooq (2015)	Clozapine prescribing in the United Kingdom: Views and experience of consultant psychiatrists	United Kingdom	Cross‐sectional survey	2771 consultant psychiatrists from the faculty of the general adult and community psychiatry of the royal college of psychiatrists. *Response rate of* 8.8% (*n* = 245).	*Workplace* Community mental health team: 50.2%; inpatient: 27.2%; crisis/home treatment team: 11.1%; rehabilitation: 10.3%; assertive outreach: 7.8%; early intervention team: 7.4%; forensic: 3.3%; others: 18.1%. Academic post: 11.7%; no academic post: 88.3%.
Qurashi et al. (2015)	An evaluation of subjective experiences, effects and overall satisfaction with clozapine treatment in a United Kingdom forensic service	United Kingdom	Cross‐sectional survey	67 patients from a forensic psychiatric hospital prescribed a stable dose of clozapine for 3 months. *Response rate of* 83.6% (*n* = 56).	—
Bogers et al. (2014)	Capillary compared to venous blood sampling in clozapine treatment: Patients' and health care practitioners' experiences with a point‐of‐care device	The Netherlands, European Union	Randomised cross‐over trial	70 patients with schizophrenia or schizoaffective disorder from mental health service North‐Holland North and mental health service rivierduinen undergoing clozapine treatment.	*Workplace* Flexible assertive community team outpatient: 44%; forensic inpatient: 23%; high‐care inpatient: 13%; recovery ward inpatient: 20%.
Davis et al. (2013)	Discontinuation of clozapine: A 15‐year naturalistic retrospective study of 320 patients	United States of America	Retrospective case note review	320 inpatients and outpatients with treatment resistant severe schizophrenia or schizoaffective disorder.	—
Cetin (2014)	Clozaphobia: Fear of prescribers of clozapine for treatment of schizophrenia	—	Narrative review	—	—
Gee et al. (2013)	Practitioner attitudes to clozapine initiation	London, United Kingdom	Cross‐sectional survey	144 clinical staff members at South London and the Maudsley national health service foundation trust.	*Workplace* Care coordinator: 1%; consultant psychiatrist: 14%; nurse: 18%; occupational therapist: 1%; pharmacy staff: 16%; psychologist: 3%; social worker: 5%; trainee psychiatrist: 42%; other: 1%. Inpatient: 63%; outpatient: 25%; unknown: 12%.
Filia et al. (2012)	Transitioning patients taking clozapine from the public to private/general practitioner shared‐care setting: Barriers and criteria	Australia	Cross‐sectional survey	60 community mental health service staff, 120 private psychiatrists registered to prescribe clozapine and 35 GPs from the bayside health clozapine general practitioner shared‐care program. *Response rate of* 46.2% (*n* = 80).	*Workplace* Community mental health service: 42.5%; private psychiatrist: 36.3%; general practitioner: 21.3%. Doctor: 70.3%; nurse: 16.2%; allied health: 12.5%.
Farhadian et al. (2011)	Fostering the use of clozapine in the severely mentally ill through academic detailing	United States of America	Cross‐sectional survey	118 mental health providers in the veterans integrated service networks 21 and 22 from the veterans health administration	—
Mistry and Osborn (2011)	Underuse of clozapine in treatment‐resistant schizophrenia	—	Narrative review	—	—
Nielsen et al. (2009)	Psychiatrists' attitude toward and knowledge of clozapine treatment	Denmark, European Union	Cross‐sectional survey	232 psychiatrists from the three highest and the three lowest clozapine prescribing counties. *Response rate of* 43.1% (*n* = 100)	*Workplace* Consultant: 73.0%; psychiatrist: 19.0%; trainee psychiatrist: 8.0%.
J. Kim et al. (2005)	Subjective response to clozapine and risperidone treatment in outpatients with schizophrenia	South Korea	Cross‐sectional survey	94 patients with stable schizophrenia treated with clozapine or risperidone at the outpatient clinic.	—
Cirulli (2005)	Clozapine prescribing in adolescent psychiatry: Survey of prescribing practice in in‐patient units	United Kingdom	Cross‐sectional study	All 83 consultant psychiatrists working in adolescent units. *Response rate of* 71% (*n* = 59).	—
Angermeyer et al. (2001)	Patients' and relatives' assessment of clozapine treatment	Germany, European Union	Semi‐structured interviews	104 clozapine treated patients with schizophrenia during inpatient or acute day‐hospital. *Response rate of* 76.9% (*n* = 80). 46 patients' relatives. *Response rate of* 26.1% (*n* = 12).	—
Waserman and Criollo (2000)	Subjective experience of clozapine treatment by patients with chronic schizophrenia	Canada	Cross‐sectional survey	168 patients from the Hamilton psychiatric hospital with chronic schizophrenia or schizoaffective disorder with at least 6 months of clozapine treatment. *Response rate* of 77.4% (*n* = 130).	*Workplace* Outpatient: 76.9%; inpatient: 23.1%.
Taylor et al. (2000)	Clozapine—a survey of patient perceptions	United Kingdom	Cross‐sectional survey	1284 patients attending 27 clozapine clinics. *Response rate of* 44.4% (*n* = 570).	—
Swinton and Ahmed (1999)	Reasons for non‐prescription of clozapine in treatment‐resistant schizophrenia	United Kingdom	Semi‐structured interviews	74 inpatients from the Ashworth hospital with significant psychotic symptoms who were never prescribed clozapine. *Response rate* of 43.2% (*n* = 32).	—
Sloan et al. (1997)	Client satisfaction in a clozapine clinic	Ireland, European Union	Cross‐sectional survey	28 patients with resistant schizophrenia attending clozapine clinics.	—

## 4 Discussion

We believe this to be the first literature review to introduce the concept of clozaphobia, in an effort to raise awareness of this issue. We hope that the end of CLZ's Risk Evaluation and Mitigation Strategy (REMS) program in the USA will contribute to the beginning of the end the unfortunate worldwide phenomenon that we propose to call clozaphobia. A summary of the analysis of clozaphobia factors, is compiled in Table 2.

**TABLE 2:** Clozaphobia: summary of barriers to clozapine prescription.

Clozaphobia: summary of barriers to clozapine prescription	
Clinician‐related barriers	Treatment‐resistant schizophrenia identification
	Indications for clozapine use
	Clinician training and confidence
	Years of experience and prescription rate
	Training and knowledge
	Lack of confidence about initiation and monitoring
	Perceptions about clozapine efficacy and prescription rates
	Knowledge about and prescription according to guidelines
	Selection of doses and therapeutic regimens
	Influence of current clinical status and clozapine as a last resort
Patient‐related barriers	Adverse effects
	Adherence to haematological monitoring
	Patient attitudes and overall adherence
	Patient capacity for cooperation
	Patient satisfaction
	Family and carer‐related attitudes.
Administrative‐related barriers	Clozapine initiation and clozapine‐specialised clinics
	Lack of resources
	The roles of different healthcare professionals
	Communication among healthcare professionals
	Lack of time and legal issues

We will make some commentaries and suggestions attempting to mitigate the clozaphobia factors already described.

Many clinicians have difficulties identifying TRS. It is essential to clarify the concept of TRS, its epidemiology and that it can appear at any stage of SCZ. Training opportunities, an exhaustive clinical history, documentation of clinical benchmarks, and the use of simpler objective scales in the initial assessment and throughout follow‐up should be promoted to help clinicians identify therapeutic response and cases of pseudo‐resistance (Agid et al. 2024; Carroll et al. 2024; Oloyede et al. 2023). Patient's age is not a contraindication to CLZ use (Agid et al. 2024; Cirulli 2005).

Some psychiatrists had never prescribed CLZ. Clinicians should assess their caseload and, if they have fewer than 20% of their patients with SCZ taking CLZ, review whether there are any more patients who meet criteria for TRS (Rubio and Kane 2019).

There is little evidence from randomised controlled trials to support antipsychotic polypharmacy effectiveness, and some studies suggest an increase in morbidity and mortality with this strategy (Agid et al. 2024; Farhadian et al. 2011). Overall, it is considered that the best currently available evidence supports the superiority of CLZ monotherapy in TRS.

Healthcare professionals need to continually update their knowledge on CLZ (Ismail et al. 2018; Oloyede et al. 2023). Programmes during residency alone may not be sufficient. Other strategies include continual medical education, development of peer‐to‐peer networks and medical advisors (Agid et al. 2024; Rubio and Kane 2019). Policymakers could take inspiration from countries like Finland and Canada, which have higher CLZ prescription rates and better patient outcomes (De Las Cuevas 2025).

Aggressive marketing of other SGAPs by pharmaceutical companies may have played a role in clinicians' reluctance to use CLZ (Mistry and Osborn 2011). One possible solution is academic detailing without commercial bias, to promote evidence‐based practices, such as key messages and unadvertisements (Farhadian et al. 2011).

A minority of clinicians consider CLZ initiation in an outpatient setting to be unsafe. However, provided that there is proper follow‐up with adequate safety guarantees, outpatient CLZ initiation is considered a safe practice and avoids the problems related to bed shortages and hospitalisation costs (Rubio and Kane 2019; Agid et al. 2024). After successful initiation of CLZ, patients should be encouraged to adopt a CLZ lifestyle, which includes dietary control, regular exercise and abstinence from substance use (Pandarakalam 2019; Opler et al. 2025).

Poor adherence to CLZ therapy should not be undervalued (Rezaie, Nazari, Safari‐Faramani, et al. 2022). Some reasons for patients' poor adherence include adverse effects and the burden of haematological monitoring. Our research found that the latter was perceived as a barrier with a frequency superior to 70% in most studies, mainly due to pain during blood sampling, the demanding nature of the monitoring schedule and limited understanding of its rationale. Still, there are already available some accessible point‐of‐care WBC analyser, virtually painless, that requires only a finger prick of blood can provide rapid results for critical decisions when it matters most (D. Kelly et al. 2020; Atkins et al. 2021). More, recently, the USA FDA's removal of the CLZ REMS is the best example that should be followed by other countries, in order to increase patients' adherence to treatment with CLZ.

A proactive search for potential adverse effects is essential because patients tend not to spontaneously report them (Mistry and Osborn 2011). Additionally, some adverse effects can interact with each other and have a cumulative negative effect, either by influencing patient's perceptions of CLZ safety (e.g., orthostatic hypotension) or by increasing the risk of more serious complications (e.g., hypersalivation leading to aspiration pneumonia).

In the absence of a life‐threatening adverse effects, the approach is usually similar: provide patient support and education and wait to see if it subsides or if toleration develops; consider dose reduction when possible and/or behavioural and pharmacological interventions (Agid et al. 2024; Gurrera et al. 2022). In the majority of cases, rechallenge after CLZ discontinuation is possible, it is often associated with a high probability of success and patients who complete 1 year of treatment are more likely to continue therapy (Agid et al. 2024; Mistry and Osborn 2011). Nevertheless, clinicians must carefully weigh the risks (e.g., possible life‐threatening adverse effects) and benefits (e.g., reduction in mortality) of CLZ treatment (D. L. Kelly and Love 2019).

Evidence shows that patients treated with CLZ are generally more satisfied than clinicians anticipate, rate their health more positively when compared to patients using other APs, and believe that the benefits of CLZ outweigh its disadvantages (Grover, Balachander, et al. 2015). Our findings are that patients are clearly more satisfied with CLZ compared other APs in general.

Some clinicians view certain patients as a lost cause or too ill to benefit from any treatment, an attitude known as therapeutic nihilism. This discourages them from formulating the diagnosis of TRS because it would do little to alter the clinical course of action. On the other hand, many patients become frustrated after having experienced multiple failed AP trials and believe that CLZ will be no different or report the stigma associated with TRS and CLZ. Stigma can make some clinicians hesitant in diagnosing TRS (Agid et al. 2024; Carroll et al. 2024; Oloyede et al. 2023). Clinicians should be trained to avoid therapeutic nihilism and stigmatising attitudes (Agid et al. 2024; Oloyede et al. 2023; Walker et al. 2024).

Some patients express reluctance toward CLZ because they are not convinced about its efficacy and advantages. Patient counselling is extremely important and, although clinicians argue that explaining CLZ treatment can be too time‐consuming, this initial investment can prove beneficial in the long run as poor patient education and insight is a significant barrier to treatment continuation (Ismail et al. 2018; Oloyede et al. 2023). Regardless of their expectations, clinicians should be encouraged and trained to establish clear communication and confidently introduce and discuss CLZ treatment with patients, providing evidence‐based information about what distinguishes CLZ from other APs. Clinicians should also be able to clarify patients about misconceptions and concerns with the treatment burden, especially concerning adverse effects, emphasising that most are mild, transient and mitigable and that severe effects are rare and, through proper monitoring, preventable (Agid et al. 2024; Oloyede et al. 2023; Rubio and Kane 2019; D. L. Kelly and Love 2019).

Some clinicians found it difficult to convince carers about CLZ initiation and that this contributed to the delay in treatment. However, it is important to involve carers because they influence patient's adherence and can assess patients' response to CLZ, as they are more aware of significant improvements in day‐to‐day symptoms and overall functioning than clinicians (Rubio and Kane 2019). Despite recognising some adverse effects, carers generally supported CLZ therapy due to its perceived benefits and the reduction in caregiver burden. Carers' attitudes and support for the patient depend on their literacy and socioeconomic status (Rezaie et al. 2022). Psychoeducation about CLZ is an important facilitator to improve CLZ prescription (Ismail et al. 2018; Oloyede et al. 2023).

Clinicians often present CLZ in a negative light, emphasising the obstacles more than its therapeutic benefits, which increases the likelihood of patients refusing it. Special emphasis should be placed on patient‐centred better outcomes associated with taking CLZ (Agid et al. 2024; Rubio and Kane 2019). Patients also tend to value clinical approaches that integrate pharmacological and non‐pharmacological interventions (Carroll et al. 2024; D. L. Kelly and Love 2019; Oloyede et al. 2023).

Useful approaches include the introduction of CLZ in short, progressive steps over several consultations while providing appealing take‐home educational materials in printed or audio‐visual format using patient‐centred language, shared decision‐making strategies, peer counselling, active collaboration in the management of the disorder, psychoeducation, family involvement and development of a trusting relationship the clinician (Agid et al. 2024; Carroll et al. 2024; Oloyede et al. 2023). Patients and carers tend to accept CLZ initiation more easily when it is presented by a senior psychiatrist (Rezaie et al. 2022). Prescribers exert the greatest influence on decision to accept medication, while care providers play a crucial role in building rapport (Carroll et al. 2024).

It should also be emphasised that some patients suffer from anosognosia, the neurological equivalent to the psychological lack of insight into their disorder (Torrey and Lieberman 2024). Patient refusal of diagnosis and theragnosis can be a relevant barrier, given that there are no approved CLZ long‐acting injectable (LAI) formulations yet. Possible solutions include combining CLZ in a single bedtime dose with a LAI AP (Agid et al. 2024). Recently, a promising CLZ transdermal patch was developed (Qadir et al. 2025).

Most general practitioners are quiet unfamiliar with CLZ (De Las Cuevas 2025). It is essential to invest in infrastructure, enhance information exchange between tertiary, secondary and primary health care (Agid et al. 2024; Blagden et al. 2020; Carroll et al. 2024; D. L. Kelly et al. 2017; Oloyede et al. 2023; Walker et al. 2024). Digital health platforms may be particularly useful in rural areas (De Las Cuevas 2025).

One of the main advantages of CLZ dispensing in community pharmacies lies in their proximity to the inhabitants of rural and remote areas. Another benefit is the friendlier and less hectic environment (Ismail et al. 2018; Wilson et al. 2019).

Pharmacists' interventions can include patient enrollment, ongoing monitoring and management of adverse effects and medication interactions, coordination of laboratory testing, clinical assessment with standardised rating scales, support for smoking cessation, patient education and adherence, dose adjustments, and interpretation of pharmacogenomic tests. Pharmacist‐run or ‐assisted CLZ clinics can lead to cost savings, high CLZ continuation rates, patient and family satisfaction and early intervention in cases of decompensation and suicidal ideation (D. L. Kelly and Love 2019).

The transition of eligible patients from tertiary to secondary, or even primary, health care can translate into better access, standardisation, flexibility, satisfaction and adherence and quality of life (Wilson et al. 2019).

Our review included a large number of survey‐based studies, some of which had a low response rate. Studies that use surveys inevitably include a risk of bias from the outset, since the subgroup of individuals who decide to take part may do so because they feel a greater interest or affinity or are more familiar with the theme. In addition, non‐responders may be those who are unfamiliar with it. The vast majority of the studies included in this review use self‐reported surveys, some of which were not anonymised, which also introduces individual bias because respondents may answer according to what they think is expected or the most correct. Consequently, given the above, the results obtained may not be generalisable, and confidence levels may be overestimated.

The vast majority of studies on this topic focus on the opinions of clinicians, mainly CLZ prescribers, with fewer studies including the opinions of other clinicians and even fewer based on the opinions of patients and carers, which could provide new perspectives on this topic. Last, but not least, there is also a wide disparity in responses depending on the geographical region in question.

## 5 Conclusion

Clozaphobia is perhaps a catchy term, though one that seems more than justified. Its broader adoption may help raise awareness of this issue, ultimately enabling more patients to access a potentially life‐changing treatment. We believe that clozaphobia contributed to the abandonment of clozapine as daily use medication in our clinical practice. We believe clozaphobia is the most important reason why there are still no higher dose pills clozapine and no LAI formulation of clozapine.

We hope that our review will encourage further research and higher‐quality studies on this topic, pushing community in order to start production of LAI formulation and higher dose pills of clozapine. Both higher dose of clozapine pills and clozapine LAI may be technically hard to produce, but they would be a very precious arsenal in our daily practice while dealing with the most severely psychotic, with anosognosia, low insight, poor treatment compliance or adherence. Imagine the power of a clozapine pill of 400 mg to be taken once a day before sleep, instead of the four pills of classic clozapine of 100 mg. Imagine the effectiveness of a LAI clozapine injection in patients already safely tested with the oral pills for months, without life‐threatening side effects.

We must paraphrase some of the most active clozapine activists. For example, Dr. Rob S. Laitman and his team, relentless activists that have been lobbying against clozaphobia, for the right of an easier and faster access to prescriptions of clozapine in the east coast of the USA. ‘Onward!’ is his motto (Opler et al. 2025). Onward against clozaphobia we must go! In conclusion, we hope this literature review regarding clozaphobia will help other scientists, clinicians, families and patients to move on, toward the direction of a more effective use of the very effective medication that is clozapine.

## Author Contributions

**Rafael Brito Castro:** methodology, formal analysis, investigation, writing – original draft preparation. **Joaquim J. Ferreira:** writing – review and editing. **João Gama‐Marques:** conceptualisation, methodology, writing – review and editing.

## Funding

The authors have nothing to report.

## Ethics Statement

The authors have nothing to report.

## Consent

The authors have nothing to report.

## Conflicts of Interest

The authors declare no conflicts of interest.

## Data Availability

The data that support the findings of this study are available from the corresponding author upon reasonable request.
